# Physical interaction with Spo11 mediates the localisation of Mre11 to chromatin in meiosis and promotes its nuclease activity

**DOI:** 10.1093/nar/gkae111

**Published:** 2024-02-26

**Authors:** Rakesh Aithal, Kuldeep Nangalia, Mario Spirek, Doris Chen, Franz Klein, Lumir Krejci

**Affiliations:** National Centre for Biomolecular Research, Masaryk University, Brno, Czech Republic; Department of Biology, Masaryk University, Brno, Czech Republic; Department of Chromosome Biology, Center for Molecular Biology, University of Vienna; Max Perutz Labs, Vienna Biocenter Campus (VBC), Dr.-Bohr-Gasse 9, 1030Vienna, Austria; Department of Biology, Masaryk University, Brno, Czech Republic; Department of Chromosome Biology, Center for Molecular Biology, University of Vienna; Max Perutz Labs, Vienna Biocenter Campus (VBC), Dr.-Bohr-Gasse 9, 1030Vienna, Austria; Department of Chromosome Biology, Center for Molecular Biology, University of Vienna; Max Perutz Labs, Vienna Biocenter Campus (VBC), Dr.-Bohr-Gasse 9, 1030Vienna, Austria; National Centre for Biomolecular Research, Masaryk University, Brno, Czech Republic; Department of Biology, Masaryk University, Brno, Czech Republic

## Abstract

Meiotic recombination is of central importance for the proper segregation of homologous chromosomes, but also for creating genetic diversity. It is initiated by the formation of double-strand breaks (DSBs) in DNA catalysed by evolutionarily conserved Spo11, together with additional protein partners. Difficulties in purifying the Spo11 protein have limited the characterization of its biochemical properties and of its interactions with other DSB proteins. In this study, we have purified fragments of Spo11 and show for the first time that Spo11 can physically interact with Mre11 and modulates its DNA binding, bridging, and nuclease activities. The interaction of Mre11 with Spo11 requires its far C-terminal region, which is in line with the severe meiotic phenotypes of various mre11 mutations located at the C-terminus. Moreover, calibrated ChIP for Mre11 shows that Spo11 promotes Mre11 recruitment to chromatin, independent of DSB formation. A mutant deficient in Spo11 interaction severely reduces the association of Mre11 with meiotic chromatin. Consistent with the reduction of Mre11 foci in this mutant, it strongly impedes DSB formation, leading to spore death. Our data provide evidence that physical interaction between Spo11 and Mre11, together with end-bridging, promote normal recruitment of Mre11 to hotspots and DSB formation.

## Graphical Abstract

**Graphical Abstract:**
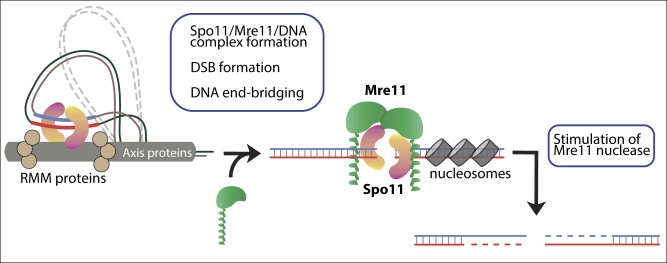

## Introduction

Meiosis is characterised by one round of DNA replication followed by two rounds of chromosomal segregation, which reduces the chromosomal content by half. At the heart of meiosis is meiotic recombination. Together with sister chromatid cohesion, cross overs physically connect homologous chromosomes to allow their accurate segregation by the end of the first meiotic division. Sexually reproducing organisms initiate recombination by the genetically programmed induction of DNA double-strand breaks (DSBs) catalysed by the evolutionarily conserved Spo11/TopoVIBL protein complex (1). DSBs have a non-random distribution along the chromosomes such that certain regions on the chromosomes, referred to as ‘hotspots’, have a higher probability of forming a DSB. Hotspot regions are not distinguished by strict DNA motifs (2–5) but by similarity to a p53 binding motif that identifies bendable DNA (6), suggesting that Spo11 recognises a state of the DNA rather than a base sequence.

Although Spo11 possesses catalytic activity, the function of at least ten other proteins is essential for DSB formation in budding yeast, establishing several layers of control over the Spo11 nuclease function. Six of these, except Mre11, were able to promote DSB formation locally when tethered to a specific chromosomal site. This implies that these factors can recruit Spo11 and suggests that they bind to Spo11 at least indirectly (7).

The Mre11–Rad50–Xrs2 (MRX) complex is essential for meiotic DSB formation and also for the resection of DNA termini at meiotic DSB sites during homology-directed repair (HDR) (8–11). Although the role of Mre11 in meiotic DNA end resection is well characterised in terms of its inherent endonuclease and 3′–5′ exonuclease activities (10,12,13), its requirement for DSB formation is still not understood.

Genetic analyses of various Mre11 mutants and truncations have helped to identify domains essential for DSB formation but not for DSB repair, and vice versa (14–17). Two separation of function alleles of *MRE11—mre11S* and *mre11-58S* bearing mutations in the conserved N-terminal region of Mre11 were proficient for DSB formation but exhibited defects in the DSB repair activity resulting in spore death (14,17). Furthermore, in the homozygous *mre11-58S* strain, Mre11 does not form a complex with Rad50 and Xrs2, thus implying that the formation of the MRX complex is not essential for the DSB formation contrary to its importance in DSB repair (16). Several studies also implicated the C-terminal domain (CTD) of Mre11 in DSB formation. Some *mre11* C-terminal alleles produced no viable spores due to abrogated DSB formation (14,16). In addition, Mre11ΔC49, lacking the last 49 amino acids, did not localise to the DSB hotspot during meiotic prophase, consistent with a role of the C-terminus in meiotic chromatin association, and showed this association of Mre11 was dependent on Spo11 (18). It was further observed that Spo11 foci extensively co-localise with Mre11 and Rad50 foci in the repair deficient *rad50S* cells suggesting possible interaction among these proteins during DSB formation and/or repair (19). However, any mechanistic understanding of the role of Mre11 in DSB formation is lacking.

Here, we discovered a direct physical interaction between Mre11 and Spo11 and characterised its role using a Mre11 truncation mutant lacking the last 29 residues *(mre11(1–663*)), strongly reducing this interaction. This allele strongly reduces its foci formation in the meiotic prophase, indicating the requirement of Mre11-Spo11 interaction for Mre11 chromatin localisation. Consistent with the loss of chromatin localisation, the *mre11(1–663*) mutant shows a defect in DSB formation and spore viability. We have also characterised a novel Mre11 mutant *mre11(1–676*) which, despite being proficient in Spo11 interaction and forming some chromatin foci, displays complete loss of spore viability. Calibrated ChIP shows an association of Mre11 to hotspots and cohesin sites in *spo11-Y135F*, which is significantly strengthened in wild-type *SPO11*, but abrogated in *spo11Δ*. This demonstrates a direct role for Spo11 in the recruitment of Mre11 to chromatin, independent of the formation of DSBs and their repair. In addition, we find that Mre11’s autonomous DNA binding activity is not required for its meiotic role. Still, all mutants that lose *in vitro* interaction with Spo11 and end-bridging activity are severely defective in DSB formation and spore viability. Interestingly, we observed that the N-terminal fragment of Spo11 can stimulate Mre11’s nuclease activity, indicating the complexity of this interaction.

## Materials and methods

### Yeast strains and plasmids

All the strains used in this study are derivatives of the SK1 background. The complete genotypes of strains are provided in Supplementary Table S1. Yeast strains were constructed by crossing with other genotypes or one-step PCR cassette replacement followed by ‘LiAc’ based transformation, using standard procedure. *Mre11-Δ668–673* deletion was generated by CRISPR/Cas9 method. Oligonucleotides and plasmids used in this study are described in Supplementary Tables S2 and S3, respectively.

### Yeast media and growth of strains

All yeast strains used in this study were grown either in solid yeast media (plates) or in liquid media YPD (2% peptone, 1% yeast extract, 2% glucose) with shaking at 200 rpm, at 30°C. For growth assays, serial dilutions of wild-type and mutant strains cultures grown overnight were spotted on YPD and YPG plates for 2–4 days at 30, 32 and 34°C (temperature sensitivity).

### Meiotic time courses

For meiotic time courses, desired diploid yeast strains were freshly streaked on YPD plates to obtain single colonies. Single colonies were then inoculated in 10 ml of YPD media in a 50 ml flask, grown overnight at 30°C, with shaking at 200 rpm. Diploid yeast cells were inoculated in a pre-sporulation medium (SPS, 0.5% yeast extract, 1% peptone, 0.17% yeast nitrogen base [Difco], 1% potassium acetate, 0.5% ammonium sulfate, 0.05 M potassium-biphthalate [pH 5.5]) to a final concentration of approximately 2 × 10^6^ cells/ml. 20–25 ml cultures were set up in 500 ml Erlenmeyer baffled flasks. Cells were grown with vigorous shaking (200 rpm) for 12–16 h at 30°C until the density reached 4 × 10^7^ cells/ml. They were then collected by centrifugation (5 min, 4000 rpm) and resuspended at a concentration of 4 × 10^7^ cells/ml in sporulation medium (SPM supplemented with amino acids [320 μl Amino acid complementation media per 100 ml SPM media]), (2% potassium acetate) and PPG (100 μl 1% PPG per 100 ml SPM), and kept shaking at 200 rpm for the whole time-course at 30°C. As the cultures were released in SPM media, this time was assigned as time t = 0 of the meiotic time course. During the meiotic progression in SPM media, samples at various time points were analysed.

### Spore viability and sporulation rate

The spore viability was analysed by dissecting the haploid spores from the diploid ascus. Diploid strains were sporulated on SPM plates (for 2 days) or SPM liquid media (2% potassium acetate) as described for meiotic cultures at 30°C overnight (or around 16–24 h). The sporulation rate of the meiotic liquid culture was scored by phase contrast microscope by scoring tetrads, triads, dyads, monads, aberrant and non-sporulating ascus. Around 200 cells/ascus were scored, and data was represented in the percentage of sporulation rate. For spore viability, tetrads were treated with Zymolyase to digest the cell walls. Sporulated yeast cells were dissolved in 192 μl of ddH_2_O, 3 μl of DTT (0.5 M) and 5 μl of Zymolyase T20 (10 mg/ml). This digestion mixture was incubated at 30°C for 30 minutes. Cells were then placed on ice, and 25–30 μl of the digested yeast cells was poured on dried YPD-rich media plates. Tetrads were dissected at a semi-automated Leitz micromanipulator. Dissected tetrads were grown at 30°C for 2–3 days until they were visible. The percentage of spore viability was scored by calculating the number of viable tetrads for 10 or 20 dissected asci.

### DAPI staining of yeast chromatin for meiotic progression

To stain the chromatin of whole yeast cells, liquid sporulating culture (100 μl) was sampled into 500 μl of 96% ethanol. The cells were then centrifuged at maximum speed for 10 secs. After completely discarding the supernatant, the cell pellet was resuspended in 50 μl of DAPI working solution (0.2 μg/ml DAPI – Sigma Aldrich). The resulting suspension was sonicated for 1 s at lower power on ice to separate the clumps of cells. To monitor the cells that progressed through the meiotic program during a time course experiment, the fraction of mono-, bi- and tetra-nucleated cells were counted (*n* = 100 cells) at indicated time points from *t* = 3 to *t* = 9 h.

### Spreading of yeast meiotic nuclei

Approximately 1 ml of sporulating culture was harvested at respective time points. The cells were pelleted down by centrifugation at maximum speed (13 000 rpm) for 10 seconds at room temperature. The pellets were resuspended in a 100 μl digestion mix per sample. This suspension was digested by incubating at 30°C for 5–10 min. The efficiency of digestion of yeast cell wall is checked on a phase contrast microscope by mixing 5 μl of digested sample and 5 μl of 1% sarcosyl detergent solution. A completely digested cell will burst open and become translucent in the phase contrast microscope after adding 1% Sarcosyl. The time of digestion of cells can be optimised. Digestion was stopped by placing the cells on ice when at least 95% of the cells were digested. To spread the digested yeast meiotic nuclei, 5 μl of digested cells were pipetted onto a clean glass slide, and 50 μl of spreading solution (2.95% (w/v) sucrose, 3.75% (w/v) paraformaldehyde, 1.0% (w/v) *N*-lauroyl-sarcosine, 1% (v/v) lipsol) was added. A clean glass pipette rolled down the resulting drop on the glass slide to make a smear on the surface of the glass slide. The slide was kept in a clear place to air dry for at least 2 h or overnight at room temperature. Once the spreads on the glass slide are dried, they are processed immediately or can be frozen at −80°C for immunostaining.

### Immunostaining of yeast meiotic spreads

The glass slides were washed in 1XPBS in a glass buffer chamber for 10 min shaking on a rotator to remove the sucrose layer. To prevent the unspecific antibody binding to the meiotic chromatin spreads, the slides were blocked for 15–20 min by blocking buffer (0.5% (w/v) bovine serum albumin, 0.2% (w/v) gelatin, from cold water fish skin in 1× PBS pH 7.25) in the humid chamber under the coverslip. The glass coverslip and blocking buffer were rinsed off by wrist flick, and slides were incubated for 2 h at room temperature or overnight at 4°C in the humid chamber with 30 μl of primary antibody diluted in blocking buffer. The coverslip was wrist flicked, and then slides were washed with 1× PBS for 10 min, shaking at room temperature. The slides were incubated with 30 μl of secondary antibody diluted in blocking buffer on a coverslip (20 × 40 mm) for 2 h at room temperature in the humid chamber. The coverslip was then rinsed off by wrist flick, and slides were washed in 1× PBS for 10 min in a glass buffer chamber with shaking. Vectashield (5–7 μl) was applied on the coverslip, and slides were flicked 2–3 times and gently placed on the coverslip to fix the yeast meiotic spreads. The coverslips were squeezed to slide by pressing the slides in the Whatman paper block. DAPI Vectashield stains the chromatin DNA of spread and has an antifade to protect against the bleaching of fluorescent conjugated secondary antibody. Immunostained meiotic nuclei spread were analysed in the fluorescent microscope or were frozen at −80°C for later analysis.

### Primary and secondary antibody

Following antibodies were used in this study: Zip1 (Raised in rabbit, Eurogenetec/Wohlrab) 1:400 dilution; Rad51 (Mouse Ab, NeoMarkers #988P1501H) 1:50 dilution; HA (Raised in rabbit, SIGMA #H6908) 1:100 dilution; Myc (Mouse IgG1, 9E10) 1:40 dilution; Hop1 (Raised in rabbit, polyclonal) 1:200 dilution; SV5-PK (Mouse IgG1, Serotec #MCA1360); Anti-rabbit (Alexa 488 conjugated, goat raised, Molecular probes #A-11034) 1:300 dilution in blocking buffer; Anti-mouse (Cy3 conjugated, goat raised, Jackson laboratories #115-165-146) 1:600 dilution in blocking buffer.

### Expression and purification of Spo11 (96–398) and other Spo11 truncations

The conserved region (20) of Spo11 containing the 5Y-CAP and Toprim domains (from amino acid residue 96 to 398) was cloned into the BamHI–SalI site of a modified pMAL-c2X plasmid to generate a fusion of MBP and 6X His-tag at the N-terminus of Spo11. It was then transformed into BL21(DE3) RIPL cells. The cells were cultured at 37°C until the OD_600_ ∼0.6, followed by induction with 1 mM Isopropyl 1-thio-β-d-galactopyranoside (IPTG) for 24 h at 12°C. Cell pellet (10 g) was resuspended in 100 ml of CBB (cell breakage buffer) (50 mM Tris–HCl pH 7.5, 10% sucrose, 10 mM EDTA, 1 mM β-mercaptoethanol (β-ME) and 0.01% Nonidet P-40 (NP40)) containing 100 mM KCl, protease inhibitor cocktail (2 μg/ml aprotinin, 5 μg/ml benzamidine, 10 μM chymostatin, 10 μM leupeptin and 1 μM pepstatin A) and 1 mM phenylmethylsulfonyl fluoride (PMSF). The cell suspension was then sonicated and clarified by ultracentrifugation (100 000 × g, 4°C, 1 h).

The supernatant fraction was then mixed with 1 ml of amylose beads (NEB) for 5 h at 4°C. The bound protein was then fractionated in batches up to 10 column volumes (CV) with 1 ml each of 20, 50, 100 and 200 mM maltose in K buffer (20 mM K_2_HPO_4_, 10% glycerol and 0.5 mM EDTA) containing 100 mM KCl, 0.01% NP40 and 1 mM β-ME. The eluted fractions were collected, loaded on a 1-ml MonoQ column (GE Healthcare), and eluted with a 15-ml gradient of 125–1000 mM KCl in K buffer containing 0.01% NP40 and 1 mM β-ME. Peak fractions were collected and concentrated using Vivaspin 20 (Sartorius) concentrators. The concentrated protein samples were stored in 10 μl aliquots at -80°C. Other Spo11 truncations described in this study were cloned, expressed and purified similarly to Spo11(96–398) protein.

### Cloning, expression and purification of Spo11–Ski8 complex

The *SPO11* gene was cloned into the BamHI-SalI sites of pETDuet-1 plasmid generating pETDuet1-*SPO11* plasmid, which adds a 6x His-tag at the N-terminus of Spo11. *SKI8* gene was cloned into the Nde1–Xho1 site to obtain pETDuet1-*SPO11*/*SKI8* plasmid fusing Ski8 with S-tag at the C-terminus and allowing co-expression with Spo11. For expression, the pETDuet1-*SPO11*/*SKI8* plasmid was transformed into BL21(DE3) RIPL cells. The cells were cultured at 37°C until the OD_600_ ∼0.6, after which the protein expression was induced by 1 mM IPTG, followed by 24 h of incubation at 12°C. Cell pellet (12.5 g) was resuspended in 90 ml of CBB. The cell suspension was then sonicated and clarified by ultracentrifugation (100 000 × g, 4°C, 1 h). The supernatant fraction was incubated at 4°C for 4 h with 0.6 ml of His-Select Ni affinity gel (Sigma). The bound protein was eluted in batches up to 10 CV with 0.5 ml each of 10, 50, 100, 200, 300 and 500 mM imidazole in K buffer containing 100 mM KCl, 0.01% NP40 and 1 mM β-ME. The peak fractions containing Spo11-Ski8 complex were stored as aliquots and used for co-precipitation assays on S-protein beads.

### Expression and purification of Mre11 and its variants

*MRE11* cloned into a pRSET-B plasmid, introducing a 6xHis tag at the N-terminus of Mre11 was a kind gift from Kunihiro Ohta. All the mutants of Mre11 used in this study were prepared by site-directed mutagenesis of this plasmid. His-Mre11 protein was over-expressed in BL21(DE3) pLysS cells by induction with 1 mM IPTG at 12°C for 24 h. After harvesting cells by centrifugation, a 10 g cell pellet was resuspended in 100 ml of CBB buffer. The cell suspension was sonicated and clarified by ultracentrifugation (100 000 × g, 4°C, 1 h). The supernatant was mixed with 1.5 ml of His-Select Ni affinity gel (Sigma) for 5 h at 4°C. The bound protein was eluted in batches up to 10 CV with 1 ml each of 10, 50, 100, 200, 300 and 500 mM imidazole in K buffer containing 100 mM KCl, 0.01% NP40 and 1 mM β-ME. Fractions containing Mre11 were pooled and loaded on 1-ml Heparin Sepharose 6 fast-flow resin (GE Healthcare). Protein fractionation was done with a 15-ml gradient of 100–1000 mM KCl in K buffer containing 0.01% NP40 and 1 mM β-ME. Peak fractions collected from Heparin resin were then loaded on a 1-ml MonoQ column and eluted with a 15-ml gradient of 125–1000 mM KCl in K buffer with NP40 and β-ME. Fractions containing Mre11 were pooled, concentrated, and stored in 10 μl aliquots at −80°C.

### DNA end-bridging assay

Double-stranded plasmid DNA (pBluescript SK {-}, 15 ng) linearised with EcoRV was incubated with indicated amounts of His-Mre11 protein in a 10 μl reaction mixture in D buffer containing 40 mM Tris–HCl pH 7.5; 50 mM KCl; 1 mM DTT and 100 μg/ml BSA. After incubation at 30°C for 30 minutes, a ligase mixture containing 10 mM MgCl_2_; 1 mM ATP and 0.5 unit of T4 DNA ligase (Fermentas) was added, and the incubation was extended for 10 minutes at 30°C. The reactions were then deproteinised by adding 2 μg/μl proteinase K and 0.5% SDS, followed by incubation at 37°C for 45 min. The samples were then analysed by electrophoresis through 0.7% agarose gel in 1× TBE buffer (0.13 M Tris, 45 mM boric acid and 2.5 mM EDTA) at 4°C and visualised by staining with SYBR-safe DNA gel stain. Gels were then scanned using the FLA-9000 Starion scanner (Fujifilm) and quantified by MultiGauge software (Fujifilm).

### Electrophoretic mobility shift assay (EMSA)

Fluorescently labeled dsDNA (49-mer, 20 nM) was incubated with the indicated amounts of His–Mre11 protein in a 10 μl reaction of buffer D. After the reactions were incubated at 30°C for 30 min, 2 μl of 6× loading buffer was added to each of the reactions, and the samples were resolved on 6% native PAGE gel in 1× TBE buffer at 4°C. Gels were then scanned using the FLA-9000 Starion scanner and quantified by MultiGauge software.

### Exonuclease assay

Indicated amounts of His-Mre11 protein were incubated with 10 nM of fluorescently labeled 5′-dsDNA substrate in D buffer containing 0.5 mM MnCl_2_ and 0.1 μg/μl BSA in a 10 μl reaction mix. The reactions were incubated at 30°C for 30 min, deproteinised by the addition of 2 μg/μl proteinase K and 0.5% SDS, followed by incubation at 37°C for 15 min. Loading buffer (2 μl) was added to each reaction, and the samples were resolved on 9% native PAGE gel in 1× TBE buffer at 4°C. Gels were then scanned using the FLA-9000 Starion scanner and quantified by MultiGauge software.

### Pull-down assay

A pull-down assay on amylose beads was employed to detect the interaction between Spo11 and Mre11 protein, taking advantage of the MBP tag fused to Spo11. Mre11 WT or Mre11 mutants (10 μg each) were incubated with 5 μg of Spo11(96–398) in K buffer containing 150 mM KCl, 0.01% NP40 and 1 mM β-ME in a 30 μl reaction mix at 30°C for 30 min. For control reactions, 10 μg of Mre11 was incubated alone in the conditions described above. After the incubation, the reaction mixture was incubated with 30 μl amylose beads (NEB) prewashed with K buffer containing 150 mM KCl and further incubated at 30°C for 30 min. The flow fractions were collected by centrifugation, and the beads were extensively washed. Loading buffer (5× loading buffer containing 0.25 M Tris–HCl pH 6.8, 0.5 M DTT, 10% SDS, 50% glycerol and 0.25% bromophenol blue) was added to the flow (F) and the bead (B) fractions, and the samples were analysed on a 12% SDS-PAGE gel followed by Coomassie blue staining.

For assays with Spo11-Ski8 complex, approximately 5 μg of Spo11-Ski8 protein was incubated with 30 μl of S-protein beads (Novagen) for 30 min at 30°C in K buffer containing 150 mM KCl. After incubation, the beads were washed, and 10 μg of Mre11 protein was added and further incubated at 30°C for 30 min. Flow fractions were collected, and the beads were washed. Loading buffer (5× SDS) was then added to the flow (F) and the bead (B) fractions, and samples were analysed on a 12% SDS-PAGE gel followed by Coomassie blue staining.

### Microscale thermophoresis

Binding affinity quantifications via microscale thermophoresis were performed using the Monolith NT.115 (Nanotemper Technologies). Affinity measurements were performed in K buffer containing 150 mM KCl, 0.01% NP40 and 1 mM β-ME. Samples were loaded into Premium capillaries (NanoTemper Technologies). Measurements were performed at 25°C, 60% Excitation power, medium MST-power and constant concentration of 25 nM fluorescently labelled Mre11 protein (Protein Labeling Kit RED-NHS secondnd Generation, NanoTemper technologies) and increasing concentration of purified Spo11 protein fragments. Data were analysed by the MO.Affinity Analysis software using initial fluorescence signal (NanoTemper Technologies).

### Calibrated ChIPSeq

Both, ChIPSeq and its calibration were performed as described previously (6), using the calibration strategy proposed by Hu *et al.* (21). In brief, HA-tagged *C. glabrata* cells were used for calibration. Query and calibration cells were crosslinked for 15 min in 1% formaldehyde at room temperature. After quenching (131 mM, glycine), cells were washed (3 × TBS) pelleted and stored on -80°C until further use. Per ChIP, 2 × 10^9^*S. cerevisiae* cells were mixed with 2 × 10^8^*C. glabrata* cells. Cell pellets were resuspended in lysis buffer (50 mM HEPAES–KOH pH 8, 140 mM NaCl, 1 mM EDTA, 1% Triton X-100, 0.1% Na-deoxycholate, 1 protease inhibitor tablet (Complete Protease Inhibitor Cocktail, Roche)), opened using FastPrep-24 5G (MP Biomedicals). After sonication (Bioruptor Pico, 20 cycles) of soluble extracts to gain 200 bp fragments an aliquot was removed as whole cell extract (WCE) to be processed without IP. The rest was incubated with antibody coupled beads (50 μl Dynabeads, Pan Mouse IgG, 3 h, 4°C), washed (3× washing buffer (50 mM HEPES–KOH, pH 8, 500 mM NaCl, 1% Triton X-100, 0.1% Na-deoxycholate, 0.1% SDS, 1 mM EDTA, 1 protease inhibitor tablet), 3× deoxycholate buffer (10 mM Tris–HCl pH 8, 250 mM LiCl, 0.5% Na-deoxycholate, 1 mM EDTA, 0.5% NP-40) and 1 × TE buffer (10 mM Tris–HCl pH 8, 1 mM EDTA), treated with RNAse (Roche) (15 min, 37°C) and incubated overnight to reverse the crosslink (1% SDS, 50 mM Tris–HCl pH 8, 10 mM EDTA, 65°C). After Proteinase K (55°C, 1 h) and the DNA was purified using QIAquick Nucleotide Removal Kit. Library preparation was performed with NEBNext® Ultra™ II DNA with NEBNext Multiplex Oligos for Illumina. AMPure® XP Beads (Beckman Coulter, Inc. A63881) were used for size selection corresponding to 100–600 bp insert size. Illumina Sequencing was performed at the VBCF NGS facility and analyzed using custom-built R-scripts (6).

## Results

### Spo11 physically interacts with Mre11

**Figure 1.**
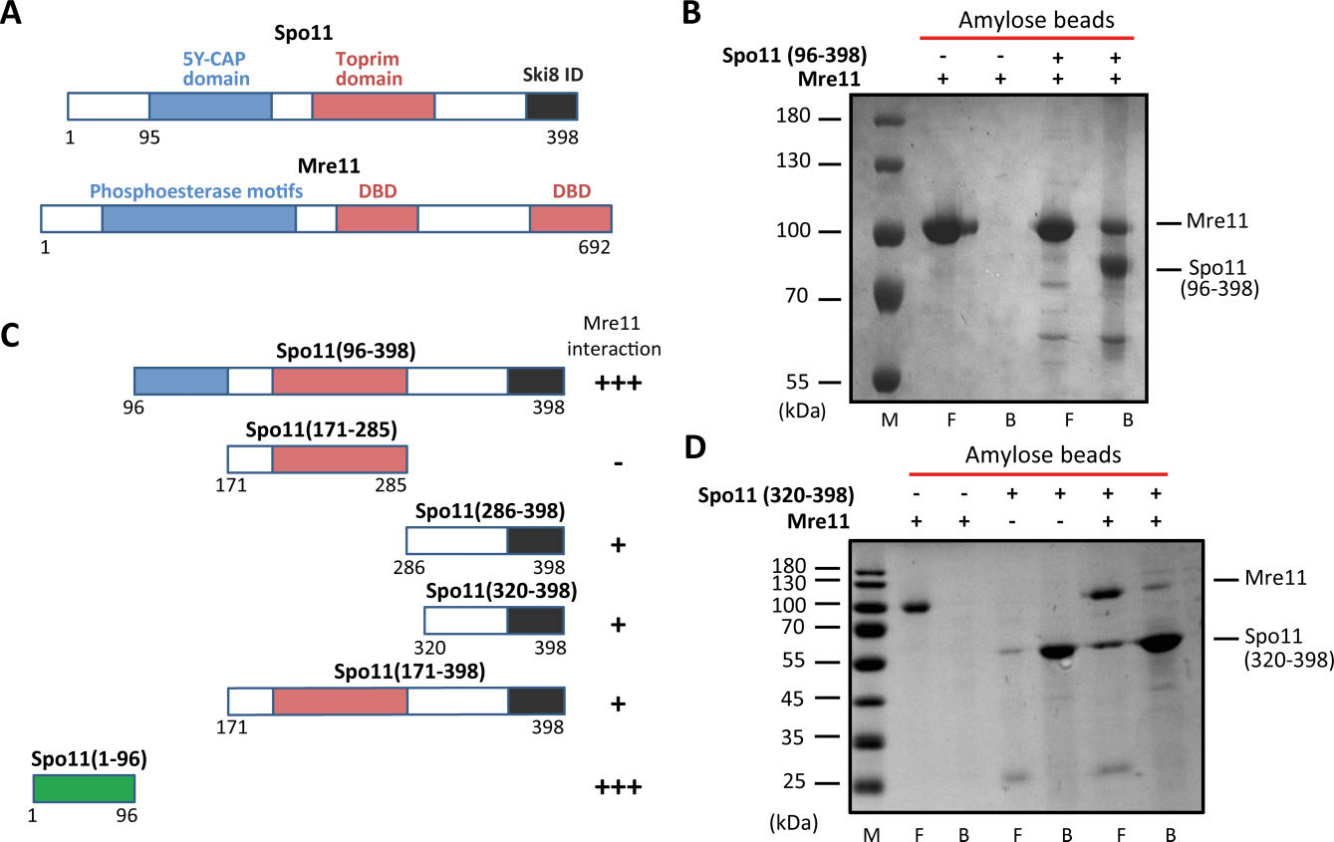
Spo11 physically interacts with Mre11. (**A**) Schematic illustration of Spo11 and Mre11 proteins. Phosphoesterase motifs, 5Y-CAP, Toprim, DNA binding (DBD) and Ski8 interaction domains (Ski8 ID) are depicted. (**B**) Mre11 (10 μg) alone or in the presence of Spo11(96–398) (5 μg) was incubated with amylose beads. The beads were washed and treated with SDS to elute bound proteins. SDS-PAGE and Coomassie blue staining of the flow (F) and bead (B) fractions. (**C**) Schematic summary of the mapping of the Mre11 interaction domain within Spo11. (**D**) Mre11 alone or in the presence of Spo11(320–398) (10 μg each) was incubated with amylose beads. The beads were washed and treated with SDS to elute bound proteins. SDS-PAGE analysed the flow (F) and bead (B) fractions followed by Coomassie blue staining.

Although Mre11 does not bind exclusively to DSB hotspots on meiotic chromatin (22), the transient association of Mre11 with DSB hotspots was shown to be Spo11-dependent (18). To test whether this association is due to direct protein interaction, we purified a conserved part of Spo11 (96–398) as a fusion with the N-terminal MBP-His tag to enhance the solubility of Spo11 protein (Figure 1A and Supplementary Figure S1A) and Mre11 as an N-terminal His-tag fusion (Supplementary Figure S1C). Pull-down with purified proteins indicates that Spo11(96–398) was able to capture Mre11 on amylose beads, while Mre11 alone did not bind to this resin (Figure 1B), demonstrating a direct physical interaction between Spo11(96–398) and Mre11.

Since Ski8 is a direct interaction partner of Spo11 during DSB formation (23), we next addressed the ability of the Spo11-Ski8 complex to interact with Mre11. We co-expressed N-terminally 6xHis tagged Spo11 with C-terminally S-tagged Ski8 protein. Purified Spo11–Ski8 complex bound to S-protein beads retained Mre11 in the bead fraction in contrast to the control reaction (Supplementary Figure S2A), confirming the interaction between Mre11 and Spo11 also in the presence of Ski8.

To map the domain in Spo11 important for the interaction with Mre11, we next generated several Spo11 truncations, including Spo11(171–285), Spo11(286–398), Spo11(320–398), Spo11(171–398) and Spo11(1–96) (Figure 1C). While the interaction with Mre11 was completely lost for the Spo11(171–285) fragment, Spo11(286–398), Spo11(320–398) and Spo11(171–398) retained a weak interaction with Mre11 (Supplementary Figure S2B-C), implying that region 320–398 of Spo11 contains an interaction domain (Figure 1D). Interestingly, the less conserved Spo11(1–96) domain strongly interacted with Mre11 (Supplementary Figure S2D), suggesting that Spo11 contains two separate Mre11 interaction domains (Supplementary Figure S2E). To corroborate these findings, we used Microscale Thermophoresis (MST) analysis, confirming the interaction of Mre11 with a set of Spo11 fragments (Supplementary Figure S2F). Spo11(96–398) exhibited the highest affinity with a *K*_d_ 5.8 ± 4.2 nM, while Spo11(1–96) displayed a *K*_d_ of 64 ± 33 nM. Accordingly, we were not able to determine the affinity value for Spo11(171–285). These results collectively pinpoint specific domains in Spo11 crucial for its interaction with Mre11.

### Spo11 regulates the DNA binding properties of Mre11

**Figure 2.**
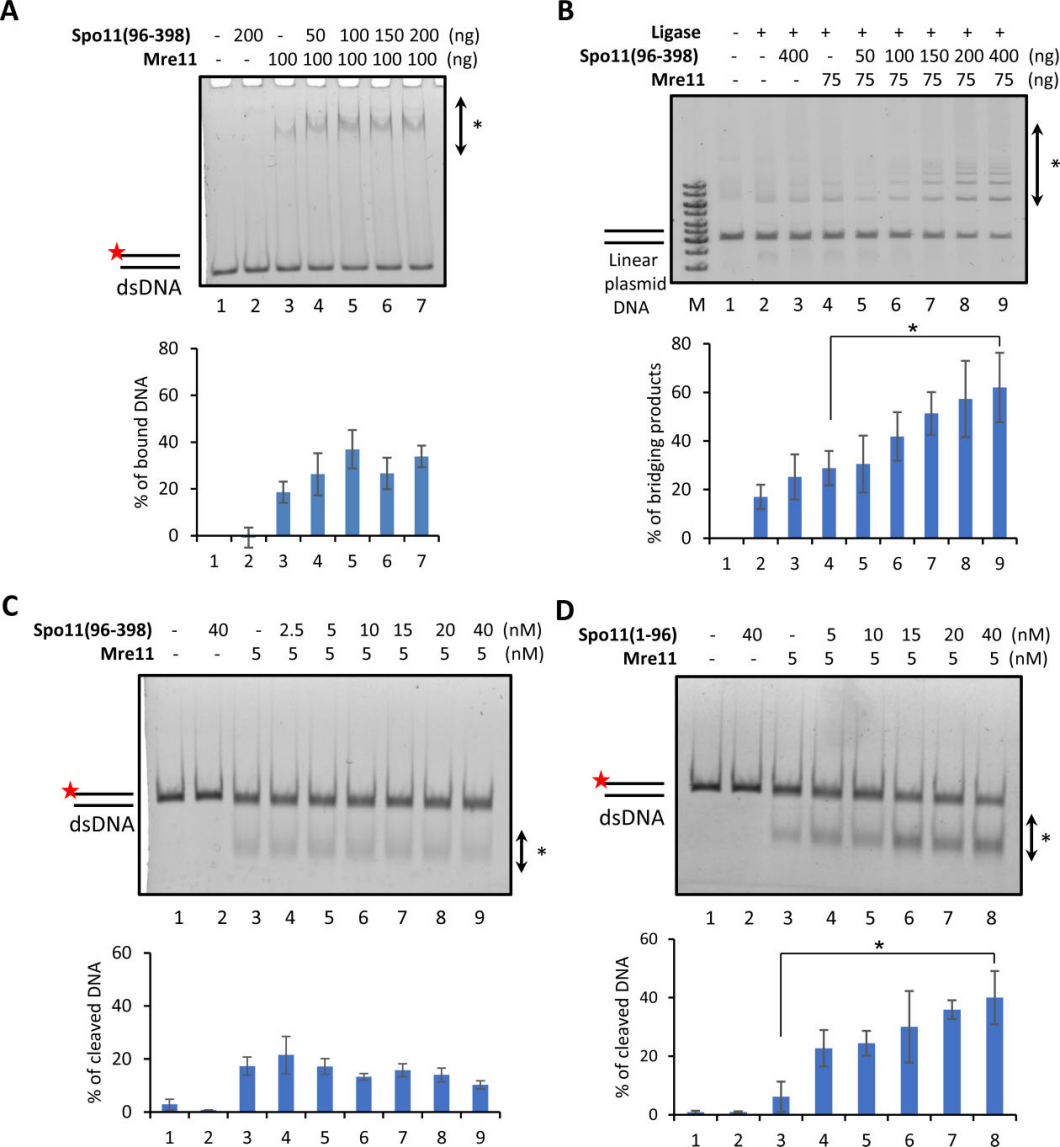
Effect of Spo11 on Mre11 activities. (**A**) Mre11 alone (lane 3) and with increasing concentration of Spo11(96–398) (lane 4–7) was incubated with fluorescently labeled dsDNA (49mer), analysed on 6% native PAGE gel, and quantified (*n* = 3). * indicates protein-DNA complexes. (**B**) Mre11 alone (lane 4) and with increasing concentrations of Spo11(96–398) (lane 5–9) were incubated with blunt end linearized plasmid in the presence of DNA ligase. Reactions were deproteinised and analysed on 0.7% agarose gel, followed by EtBr staining. After quantification, the values were normalized to the ligase control (lane 2) and plotted; data are means ± s.d. (*n* = 3) * indicates the bridging products. (**C**,**D**) Mre11 alone (lane 3) and with increasing concentrations of Spo11(96–398) (C, lane 4–9, (n = 2–5)) or Spo11(1–96) (D, lane 4–8, (*n* = 3)) were incubated with fluorescently labeled dsDNA (49mer) to assess the exonuclease activity in the presence of MnCl_2_. After incubation, reactions were deproteinised, analysed on 9% native PAGE gel and quantified. P-values are obtained by Student's *t*-test (two-tailed): * *P* ≤ 0.05. * indicates products of the Mre11 nuclease activity.

The identification of direct physical interaction between Spo11(96–398) and Mre11 prompted us to test the effect of this interaction on Mre11 activities. We first employed an electrophoretic mobility shift assay (EMSA) with a fluorescently labeled dsDNA substrate to test the impact of Spo11(96–398) on the DNA binding of the Mre11 protein. Reactions with Mre11 alone showed a prominent band shift corresponding to a specific protein–DNA complex (Figure 2A). Next, a sub-saturating concentration of Mre11 (100 ng) was chosen to test the effects of Spo11. The addition of Spo11(96–398) increased two-times the binding of both proteins to dsDNA and slightly shifted the mobility of protein-DNA complexes (Figure 2A), indicating that Spo11(96–398) enhances/or stabilises the DNA binding activity of Mre11.

Mre11 was previously reported to bind to DNA ends and to promote the ligation of two DNA molecules by DNA ligase by bridging the ends between the DNAs (24). To observe if Spo11(96–398) can stimulate this Mre11 activity, we incubated Mre11 alone and with increasing concentrations of Spo11(96–398). The addition of Spo11(96–398) showed a robust, dosage-dependent enhancement of Mre11-mediated end-bridging activity. In contrast, Spo11 did not affect ligase activity in the absence of Mre11 (Figure 2B), indicating that Spo11 helps Mre11 hold the broken ends together for further processing.

Since Mre11 also possesses an exonuclease activity (12,13), we next tested the effect of Spo11(96–398) on the cleavage of fluorescently labeled dsDNA substrate. However, no difference in the Mre11 nuclease activity was observed in the presence of Spo11(96–398) (Figure 2C). Interestingly, the addition of Spo11(1–96) that carries another Mre11 interaction domain resulted in robust stimulation of Mre11 exonuclease activity (Figure 2D), suggesting Spo11 could also play a role in aiding DSB repair and that the two Mre11 binding domains within Spo11 might have separate functions. We also wished to test the effect of Mre11 on Spo11 activities reciprocally, but we have not been able to reconstitute the *in vitro* endonuclease activity either with Spo11 alone or in complex with Ski8 (data not shown).

To unravel the possible interplay between separate Spo11 fragments and their impact on Mre11 activities, we individually assessed Spo11(1–96) and Spo11(96–398), both alone or in combination. The data revealed a compelling dynamic: Spo11(96–398) exerted a dominant negative effect on the stimulatory action of Spo11(1–96) regarding Mre11 exonuclease activity (Supplementary Figure S3A). Conversely, Spo11(1–96) exhibited a dominant negative effect on Spo11(96–398) in its ability to stimulate Mre11-mediated end-bridging activity (Supplementary Figure S3B). These results indicate a complex interplay within the Mre11 and Spo11 interaction, suggesting intricate regulation of this interaction.

### The C-terminal domain (CTD) of Mre11 is required for its interaction with Spo11

**Figure 3.**
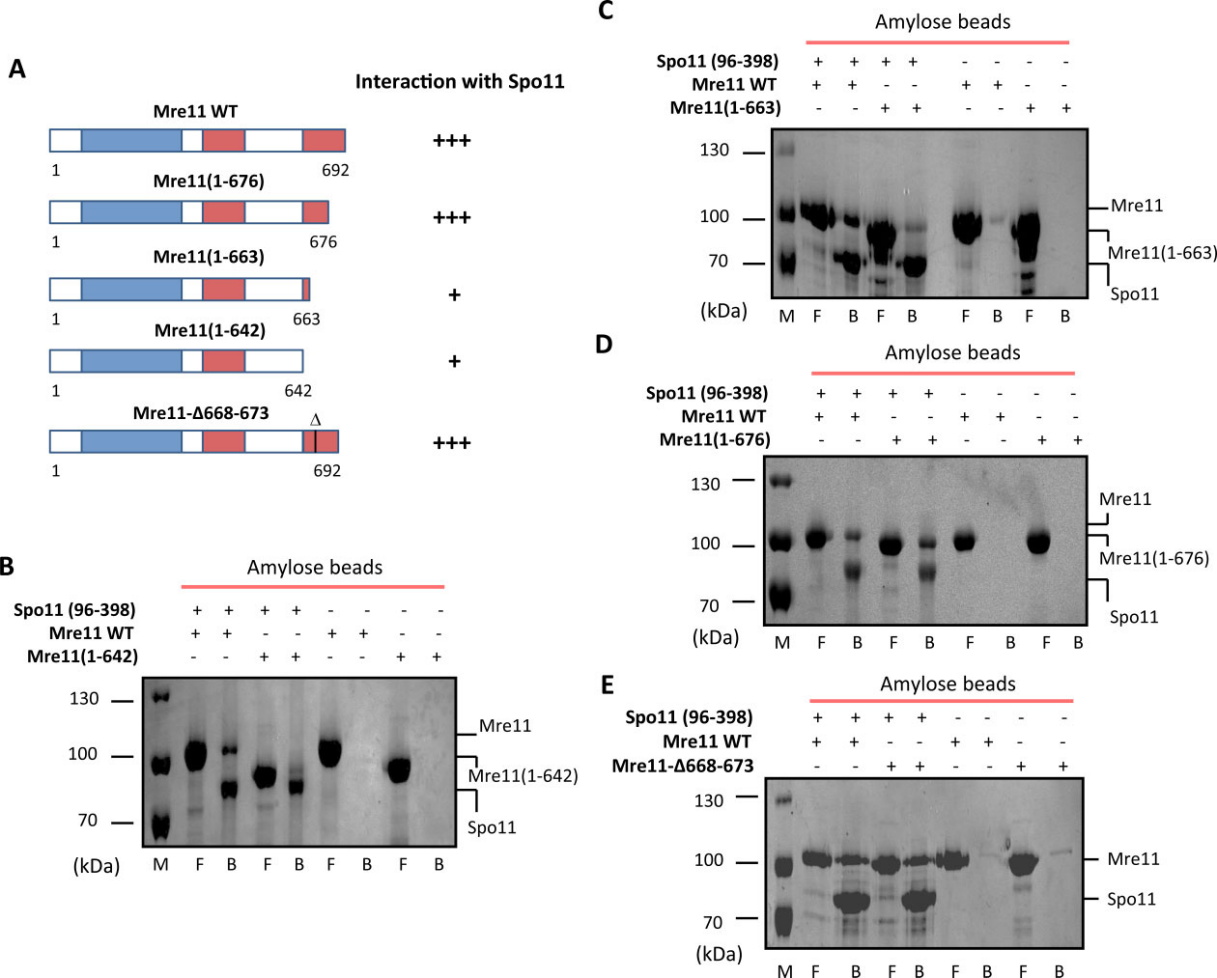
Mapping Mre11 region required for Spo11 interaction. (**A**) Schematic summary of mapping the Spo11 interaction region within Mre11. Phosphoesterase motifs and DNA binding domains are depicted as blue and red rectangles, respectively. Full-length Mre11 WT, Mre11(1–642) (**B**), Mre11(1–663) (**C**), Mre11(1–676) (**D**) and Mre11-Δ668–673 (**E**) alone (10 μg each) or in the presence of Spo11(96–398) (5 μg) were incubated with amylose beads. The beads were washed and treated with SDS to elute bound proteins. The flow (F) and bead (B) fractions were analysed by SDS-PAGE, followed by Coomassie blue staining.

To map the Spo11 interaction domain within Mre11, we took advantage of the previously demonstrated essential role of Mre11 CTD to initiate meiotic recombination by mediating DSB formation (14–16). Therefore, we generated a set of Mre11 C-terminal truncations to test their binding to Spo11 (Figure 3A). Pull-down assays using Spo11(96–398) and individual Mre11 truncations showed an apparent reduction of interaction for the Mre11(1–642) and Mre11(1–663) fragments, while Mre11(1–676) retained the full ability to interact with Spo11 (Figure 3B–D). These data suggest that the interaction with Spo11 requires 13 of the last 29 amino acid residues of the Mre11 protein and could contribute to the severe meiotic phenotypes of various C-terminal *MRE11* deletion alleles.

### DNA binding activity of the Mre11 truncations

In addition to facilitating interaction with Mre11, the Mre11 CTD has also been shown to possess a DNA binding activity (15,16). Therefore, we characterised the DNA binding abilities of individual Mre11 truncations using EMSA with dsDNA substrate. All C-terminal Mre11 truncations show a defect in DNA binding of variable magnitude (Supplementary Figure S4F). Compared to Mre11-WT, which forms a stable protein-DNA complex, Mre11(1–642) and Mre11(1–663) exhibit severe defects in DNA binding activity (Supplementary Figure S4A-C). Interestingly, the Mre11(1–676) fragment showed a reduction in the proportion of DNA–protein complex compared to Mre11 WT but also shifted to different mobility than the wild-type (Supplementary Figure S4D), indicating a formation of the altered complex with DNA. These data show that the region critical for DNA binding is localised between positions 663 and 676 and suggests that the last 16 amino acid residues of Mre11 are important for a stable association of Mre11 with the dsDNA. Therefore, we constructed a small internal deletion within Mre11 (Δ668–673), eliminating the basic six amino acid patch expected to be involved in DNA binding. This truncation shows a marked reduction in the DNA binding activity comparable to Mre11(1–663), but with the appearance of only multiple weak bands suggesting some unstable interaction with DNA (Supplementary Figure S4E). Notably, the interaction with Spo11 is not affected in Mre11–Δ668–673 (Figure 3E), indicating that this region is necessary only for DNA binding. Therefore, Mre11–Δ668–673 represents a new separation of function mutant, providing a tool to characterise the consequences of DNA binding defects of Mre11 on meiosis.

We next tested the nuclease activity of these Mre11 C-terminal truncations. Mre11(1–663) and Mre11–Δ668–673 are severely reduced for exonuclease activity, corresponding to their defect in DNA binding (Supplementary Figure S5A and C). Similarly, Mre11(1–676), with an altered ability to bind DNA, shows a prominent 8-fold reduction in its nuclease activity compared to the wild-type protein (Supplementary Figure S5B and D). The correlation of defect in DNA binding and nuclease activities suggests that DNA binding within the CTD domain is required for Mre11 exonuclease activity *in vitro*. Given our previous observation of Spo11 (1-96) stimulating Mre11 nuclease activity, we next tested this effect with Mre11(1–676). Surprisingly, while higher concentration of Mre11(1–676) were required, we observed an unexpected inhibition of nuclease activity in the presence of Spo11 (1–96) (Supplementary Figure S5E). Notably, despite any observable defect in the direct interaction between this Mre11 fragment and the N-terminus of Spo11 (Supplementary Figure S5F), this data indicates a possible loss of the Spo11 interaction-induced modulation of Mre11 nuclease activity.

### DNA end-bridging activity of Mre11 mutants

**Figure 4.**
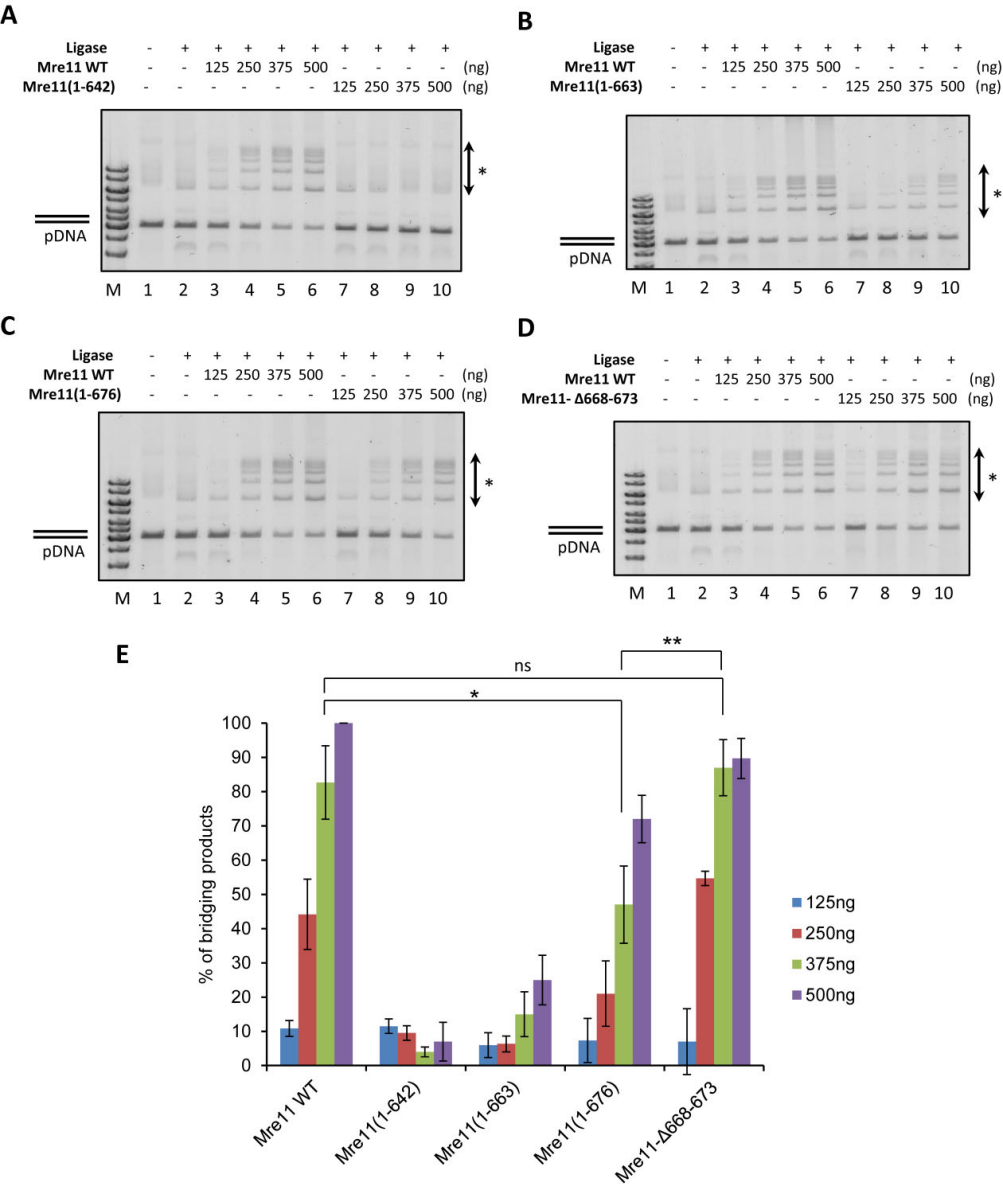
DNA end-bridging activity of Mre11 variants. (**A**) Increasing concentrations of Mre11 (lane 3–6) or Mre11(1–676) (lane 7–10) were incubated with blunt end linearized plasmid in the presence of DNA ligase. Reactions were deproteinised, analysed on 0.7% agarose gel and stained with EtBr. (**B**) Mre11(1–663), (**C**) Mre11(1–642), (**D**) Mre11-Δ668–673 were analysed as in panel (A). * indicates the end-bridging products. (**E**) Quantification of three independent experiments normalized to the ligase control (lane 2); data are means ± s.d. *P*-values are obtained by Student's t-test (two-tailed): ns *P* > 0.05; * *P* ≤ 0.05; ** *P* ≤ 0.01. All assays were done in triplicates except data for Mre11 WT *n* = 6.

Next, we asked if the CTD has any role in the end-bridging activity of Mre11. Similarly to the DNA binding activity, Mre11(1–642) and Mre11(1–663) showed robust and Mre11(1–676) slight reduction in the end-bridging compared to the wild-type protein (Figure 4A–E). Surprisingly, wild-type levels of end-bridging activity were observed for the Mre11–Δ668–673 mutant, despite its severe defect in DNA binding (Figure 4D). In summary, our results highlight the indispensable role of the MRE11 CTD in both DNA binding and end-bridging activities. Notably, these activities are distinct and independent, featuring a crucial DNA binding domain located within the 668–673 region and an end-bridging domain situated distally from the residue 673.

### Meiotic progression and spore viability of Mre11 mutants

To characterise the meiotic phenotypes of *mre11(1–663)*, *mre11(1–676)*, and *mre11-Δ668–673* mutants, we created the respective deletions at their native chromosomal locus to ensure the expression of mutant proteins from their endogenous promoters. All Mre11 mutants exhibited normal vegetative growth (Supplementary Figure S6A) and showed expression levels comparable to the WT strain (Supplementary Figure S6B and data not shown). Meiotic progression of all mutants was assessed by DAPI staining of meiotic chromatin at different time points. Importantly, the first meiotic division occurred on time (blue descending line), indicating no major delay (Supplementary Figure S7A–D) that would be indicative of an activated DNA damage checkpoint. A slight delay was observed for a subset of cells in the *mre11(1–663)* mutant (Supplementary Figure S7C), possibly reflecting its repair defect in addressing certain spurious S-phase related damage. Subsequently evaluation of spore viability revealed that while *mre11-Δ668–673* exhibited normal spore viability, no viable spores were found in the *mre11(1–663)* and *mre11(1–676)* strains (Supplementary Figure S8B). To explore the potential compensatory role of the prominent yeast exonuclease Exo1 in the context of the defective exonuclease function of mre11*-Δ*668–673, we deleted Exo1 in both the MRE11 WT or mre11*-Δ*668–673 backgrounds. Meiotic progression and spore viability were then assessed. This analysis exposed synthetic defects caused by *mre11-Δ668–673* in the *exo1Δ* background, manifesting as an additional one-hour delay in meiotic divisions and a further reduction in the yield of viable spores - from 62% in *exo1Δ* to 29% in the double mutant (Supplementary Figure S8). These findings support an interpretation in which Exo1 contributes to the compensation for the loss of Mre11’s *in vitro* exonuclease activity.

### DSB formation in Mre11 mutants

**Figure 5.**
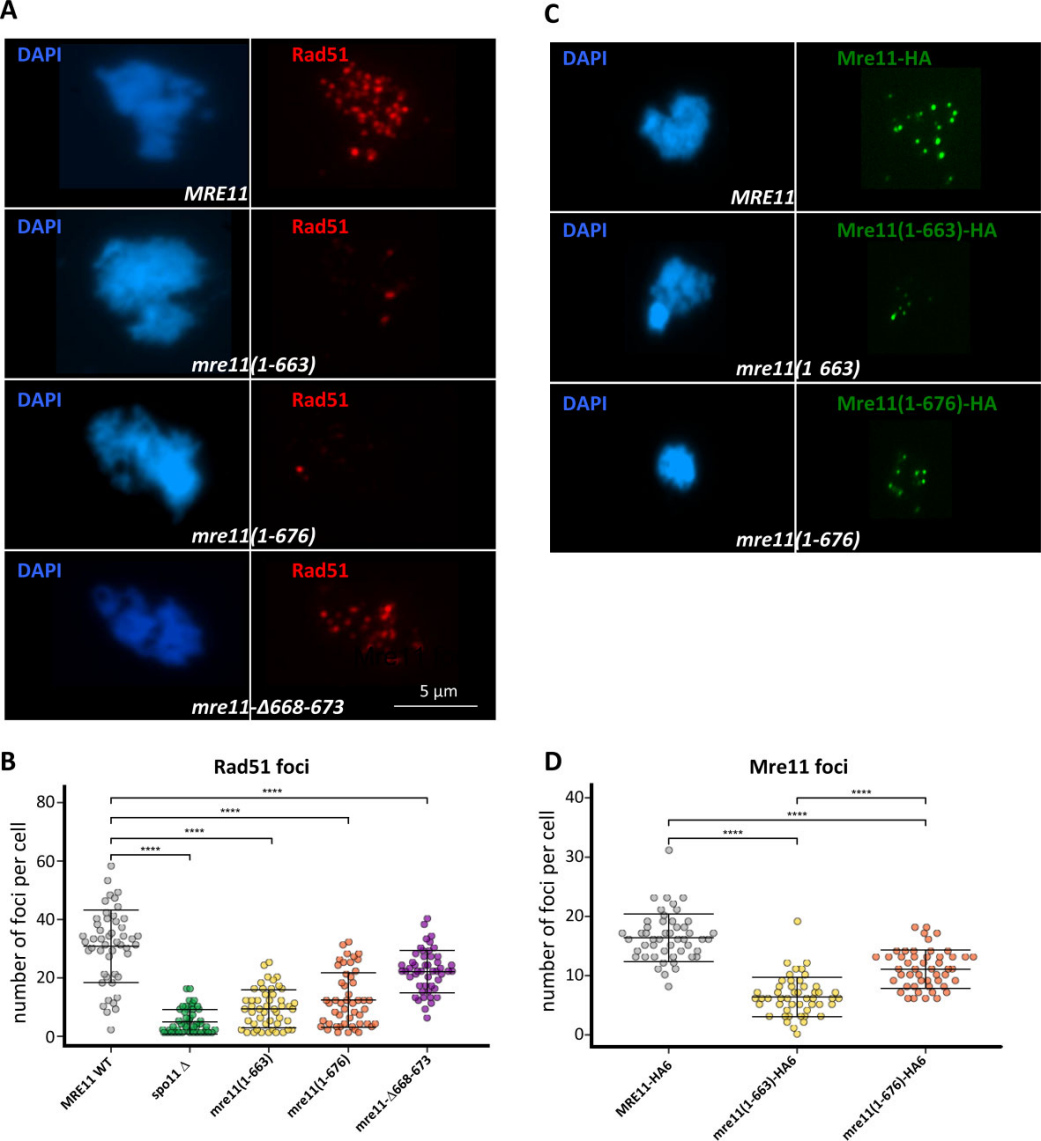
Recombination foci in Mre11 variants. (**A**) Rad51 foci formation in Mre11 mutant strains. Meiotic nuclear spread of sporulating wild-type, *mre11-Δ668–673*, *mre11(1–663)*, and *mre11(1–676)* strains at 4 h after transfer to SPM media, stained with DAPI and anti-Rad51 antibody. (**B**) Quantification of Rad51 foci from panels. Fifty images per strain were analysed, and the Man-Whitney test was used for significance and *P*-value calculation. (**C)** Mre11 foci formation and chromatin association. Meiotic nuclear spread of sporulating wild-type, *mre11(1–663)*, and *mre11(1–676)* cultures at 4 h after transfer to SPM media, stained with DAPI and anti-HA antibody. (**D**) Quantification of Mre11-HA foci from panels (A–C). Fifty images per strain were analysed, and the Man–Whitney test was used for significance and *P*-value calculation.

One reason for high spore lethality but relatively normal meiotic progression could be the inability to induce or process meiotic double-strand breaks (DSBs). Resection of meiotic DSBs promotes the formation of ssDNA, followed by induction of RPA, Rad51 and Dmc1 foci. Therefore, as a proxy of normal DSB formation and processing in meiotic nuclear spreads, we monitored the foci formation of two early recombination proteins: Rfa2, a sub-unit of RPA protein, and the Rad51 recombinase (Figure 5A and Supplementary Figure S9A). Rad51 and Rfa2 foci were reduced in *mre11(1–663)* and *mre11(1–676)* strains to levels similar to *spo11Δ* (Figure 5B, Supplementary Figure S9B and C), indicating a lack of DSB formation consistent with the complete absence of spore viability. Mre11 null mutants also do not form meiotic DSBs (25). On the other hand, *mre11-Δ668–673* exhibited substantial levels of Rad51 foci (Figure 5A and B), demonstrating sufficient DSB formation consistent with the normal spore viability observed in this mutant. We note that the ability of *mre11(1–676)* to bind to Spo11 did not guarantee DSB formation, with Spo11 binding being necessary but not sufficient.

### The association of Mre11 with chromatin

**Figure 6.**
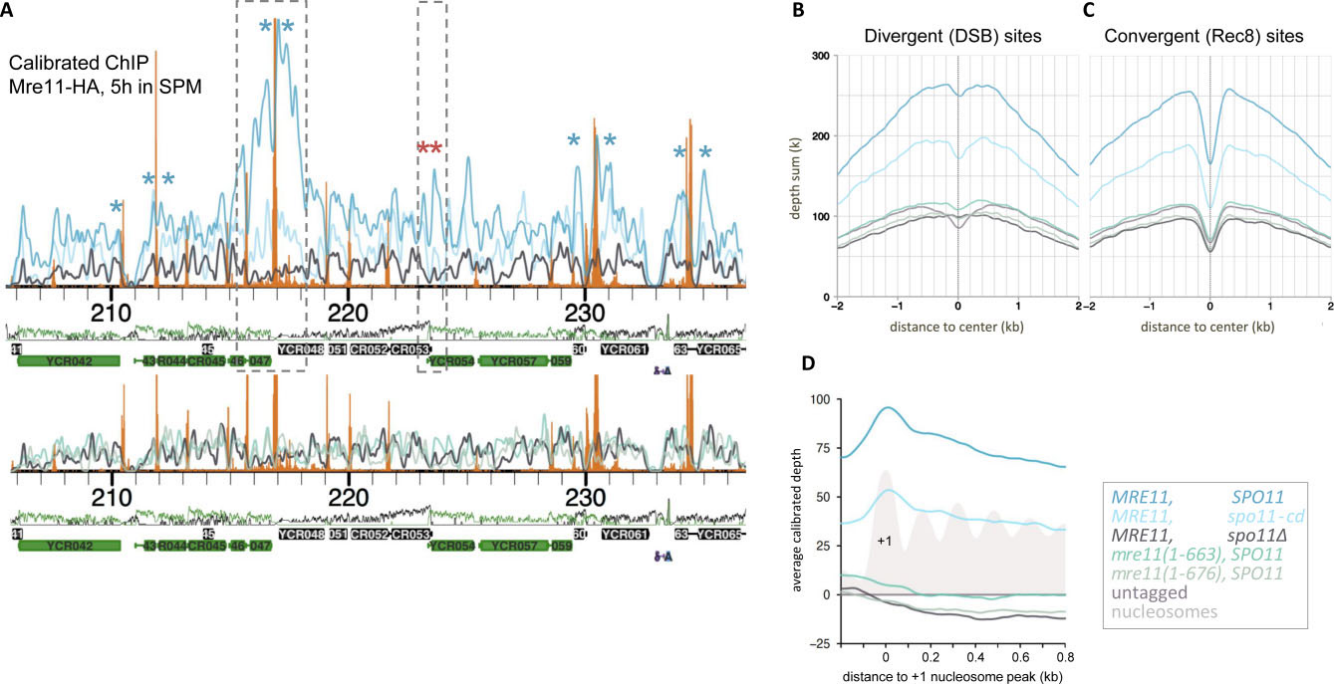
Spo11 promotes accumulation of Mre11, but not of *mre11(1–663)* or *mre11(1–676)* to hotspots and core sites. (**A**) Upper panel: ChIPSeq analysis of Mre11-HA6 in wild-type *SPO11* (blue), *spo11Δ* (black) and catalytically dead Spo11 (*spo11-cd*, light blue) backgrounds. Orange bars mark DSB positions (49), blue and red asterisks highlight Mre11 peaks adjacent to DSB sites and to a site of convergent transcription, respectively. Dashed rectangles mark the width of a hotspot and the cohesion site. Lower panel: ChIPSeq of *mre11(1–663)* (blue-green), *mre11(1–676)* (light grey), untagged (dark grey). All profiles calibrated to each other. (**B**) Aggregation of Mre11-HA signals from all divergent transcription sites centered on midpoints. (**C**) Aggregation of Mre11-HA signals from all convergent transcription sites centered on midpoints. (**D**) Average of Mre11-HA and nucleosome signals (4 h in meiosis (27)) aligned at the +1 nucleosome peak over all ORFs with a detectable nucleosome peak within ±50 bp of the annotated ORF start (2064 ORFs).

The C-terminal 49 amino acids of Mre11 were shown to be necessary for binding to hotspots and DSB formation (18). To ask whether the defective DSB formation of our mutants is due to their inability to associate stably with chromatin, we tagged *MRE11 WT* and individual mutants with 6x HA-tag at their C-terminus. Mre11 foci were observed on meiotic nuclear spreads at 4 h after transfer to the sporulation medium (Figure 5C). A drastic reduction of Mre11 foci (around 30% of WT levels; Figure 5D) was observed for the Spo11 interaction defective allele (*mre11(1–663))*, and a mild reduction (around 80% of WT; Figure 5D) was detected for the *mre11(1–676)* mutant with regular Spo11 binding. For more in-depth analysis, we performed calibrated ChIPSeq with Mre11-HA6 in wild type, *mre11(1–663)* and *mre11(1–676)* strains. Indeed, Mre11 signals associate with DSB hotspots, confirming earlier results (18). However, our current precise knowledge of DSB sites (6,26), revealed that Mre11 tends to closely flanks strong DSB sites, with a reduced signal at the actual cut site (Figure 6A, blue asterisks). As reported earlier (22), Mre11 also binds to many other positions, including cohesin sites (Figure 6A, red asterisks), situated at sites of convergent transcription in budding yeast, while the strongest hotspots are found at sites of divergent transcription. Aggregated analysis over the complete genome (Figure 6B and C) show that Mre11 signal accumulates at these two features. The robust correlation of Mre11 signals with meiotic Top2 signals (Supplementary Table S4), which closely mirror meiotic hotspots (6), further underscores Mre11’s affinity to DSB sites. We also noted a detectable positive correlation with nucleosomes (27). Figure 6D shows that Mre11 avoids the nucleosome-depleted region upstream of ORFs and correlates specifically with the + 1 nucleosome. This observation is consistent with the physical interaction of Mer2 with both, nucleosomes and Mre11 (28). To elucidate the impact that Spo11-dependent DSBs on recruitment of Mre11, we quantitatively compared the Mre11 patterns between the *SPO11, spo11Δ* and *spo11-cd* (*spo11-Y135F*) backgrounds. Importantly, Mre11 does not bind to chromatin in the complete absence of Spo11, resulting in a ChIP pattern indistinguishable from the untagged control. In contrast, even with elimination of DSBs in the *spo11-cd* background, there is still significant Mre11 binding (Figure 6A–D), albeit at reduced levels compared to *SPO11* wild-type. This underscores a non-catalytic role of the Spo11 protein in the recruitment of Mre11 to chromatin and positions Mre11 immediately adjacent to DSB hotspots independent of meiotic DSB formation, a prerequisite for a protein essential to this function. It also argues, that Mer2 is not sufficient to recruit Mre11 to chromatin *in vivo*. Contrary to expectations, we found that DSBs contribute to normal Mre11 levels even at chromosomal locations other than hotspots, suggesting that a nucleus-wide response, such as DNA damage signaling may contribute to wild-type Mre11 distribution patterns. Both C-terminal truncations, *mre11(1–663)* and *mre11(1–676)*, eliminated all chromosomal ChIP signals, consistent with their inability to promote DSB formation. Our findings highlight the significance of the Spo11–Mre11 interaction in the localisation of Mre11 to chromatin for DSB formation, hinting on the possible role of nucleosomes in this process.

### Axis formation is defective in Mre11 mutants

**Figure 7.**
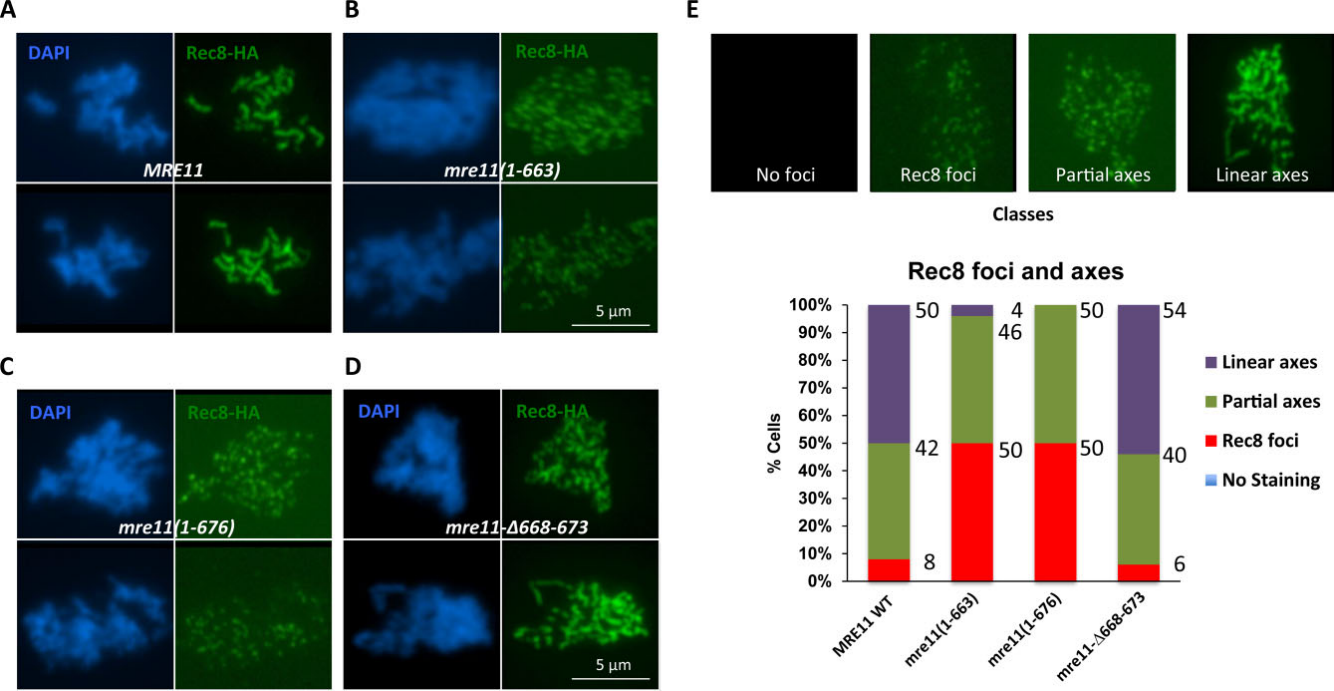
Rec8 foci and axis formation in Mre11 variants. Meiotic nuclear spread of sporulating wild type (**A**), *mre11-Δ668–673* (**B**), *mre11(1–663)* (**C**) and *mre11(1–676)*, and *mre11-Δ668–673* (**D**) cultures at 4 h after transfer to SPM media, stained with DAPI and anti-HA antibody for Rec8. (**E**) Upper four images represent typical examples for the categories analysed. Bars show the frequency of nuclei falling into those categories for the experiment shown in panels (A–D). Fifty images were analysed per strain.

DSB formation occurs in a unique chromosome architecture where the chromatin is organised into a linear array of loops held together by an underlying proteinaceous axis, known as the axial element (29,30). The axial element in yeast contains the axis proteins Red1 and Hop1 (31,32), and the meiotic cohesin complex with a meiosis-specific kleisin subunit Rec8 (33). Therefore, we next tested axis formation in our Mre11 mutants by monitoring the chromatin association of Rec8 and Red1 proteins. In WT cells Rec8 produces continuous staining extending over the length of each chromosome during pachytene (Figure 7A and E) (33,34). Similarly, long axes were also observed in the *mre11-Δ668–673* mutant (Figure 7D and E), showing that axis formation is largely independent of the Mre11 CTD DNA binding domain. In contrast, Rec8 fails to form long axes in both *mre11(1–663)* and *mre11(1–676)* mutants but instead remains in the form of discontinuous foci (Figure 7B, C and E) corresponding to short, unpaired axial element fragments. Also, the Red1 protein extends over the whole length of meiotic chromosomes in the WT strain (Supplementary Figure S10A) (32). Similar to Rec8, Red1 remains in discontinuous foci in both *mre11(1–663)* and *mre11(1–676)* strains (Supplementary Figure S10B and C), which correspond to the short axial element fragments also observed in *mre11Δ* (14,17) and in general for mutants unable to induce meiotic DSBs.

We also monitored the levels of synapsis in these mutants by staining for the central element protein Zip1. In WT and the *mre11-Δ668–673* mutant strains, Zip1 forms linear axes along the length of the paired homologous chromosomes (Supplementary Figure S11A and B), indicating that complete synapsis occurs at normal frequency. In contrast, *mre11(1–663)* and *mre11(1–676)* strains show no synapsis but the formation of aberrant Zip1 aggregates known as polycomplexes (Supplementary Figure S11A and B). All cytological observations reported above for these two mutants are expected consequences of their inability to form DSBs. In summary, these observations indicate that although the Mre11 CTD is crucial for DSB formation and chromatin binding of Mre11, the *in vitro* DNA binding and resection defects of Mre11–Δ668–673 do not compromise chromosome pairing and synapsis *in vivo* and are compensated for in meiosis.

## Discussion

### Spo11–Mre11 interaction is likely necessary but not sufficient for Mre11 chromatin association

The C-terminal 49 amino acids of Mre11 were previously implicated in the association of Mre11 with chromatin (16,18). Therefore, we predicted a direct physical interaction between Mre11 and Spo11 to recruit Mre11 to chromatin. Indeed, we identified a direct interaction between Mre11 and a soluble Spo11 fragment (96–398) *in vitro* and mapped the region required for the interaction to the last 29 amino acids of Mre11 (Supplementary Table S5). Deleting this domain in *mre11(1–663)* leads to loss of Spo11 interaction, loss of chromatin binding and loss of DSB formation *in vivo*. Interestingly, deleting only 16 amino acids in the *mre11(1–676)* mutant caused loss of all ChIP-signal and DSB formation, despite retaining its ability to interact with Spo11 *in vitro*, as well as the formation of most foci. The *mre11(1–676)* mutant thus represents a novel separation of function allele for Mre11. The fact that *mre11(1–676)* binds Spo11 normally, yet Mre11 fails to localise *in vivo* to chromatin, exposes the role of the last 16 amino acids in stabilising the Mre11 chromatin interaction. In addition, the inhibition of Mre11(1–676) nuclease activity in the presence of Spo11 (1–96) underscores the dynamic nature of the Mre11-Spo11 interaction and adds another layer of complexity to our understanding of their collaborative function during DSB formation.

### Axis and SC formation in Mre11 mutants

Axial element proteins Hop1 and Red1 are required for normal levels of DSBs (35,36) at Red1- and Hop1-rich genomic regions (37,38). The correct distribution of these proteins depends on the meiosis-specific kleisin subunit Rec8 (38,39). We observe Rec8 and Red1 foci corresponding to short axial element fragments in *mre11(1–663)* and *mre11(1–676)* mutants, as predicted in the absence of DSBs, because continuous axes and synapsis depend on the formation of DSBs in the yeast *S. cerevisiae*. The Zip1 protein binds meiotic chromosomes specifically at synapsed regions (40), thus quantifying the formation of the synaptonemal complex (SC). That synapsis is completely lacking in *mre11(1–663)* and *mre11(1–676)* mutants provides sensitive evidence for the absence of any DSBs.

### The DNA binding and *in vitro* exonuclease activities of Mre11 are not essential for meiosis in yeast

Mre11 CTD was also shown to possess a DNA binding domain (15,16), but its relevance in the meiotic functions of Mre11 was unclear. We showed that the six amino acid deletion mutant *mre11–Δ668–673* has severe defects in DNA binding and in its exonuclease activity *in vitro*. On the other hand, this mutant showed normal interaction with Spo11 and DNA end-bridging activity (Supplementary Table S5). Surprisingly, the *mre11–Δ668–673* by itself did not cause meiotic defects *in vivo*, being normal for Rad51 foci numbers, axis development, SC formation, meiotic progression, spore formation and viability, indicating normal DSB formation and processing. This suggests that the autonomous DNA binding activity of Mre11’s CTD is not essential in meiosis, and Mre11 can be recruited to chromatin by protein-protein interactions associated with the Mre11 CTD. The mutation does, however, aggravate the meiotic defects of an *exo1Δ* mutation, showing that Exo1 partially compensates for defects of *mre11–Δ668–673*. Interestingly, while the endonucleolytic function of Com1/Sae2 and MRX is irreplaceable, it seems that the following exonuclease activity can be performed by any of several cellular activities. The fact that *mre11–Δ668–673*, which doesn’t bind DNA *in vitro*, shows normal chromosomal recruitment and DSB formation, while *mre11(1–676)*, which binds DNA *in vitro* reasonably well fails to bind *in vivo*, indicates that the last 16 amino acids are required for protein-protein interaction-mediated recruitment to the chromosome. The observed, Spo11-dependent preference of Mre11 for the + 1 nucleosome may be related to its interaction with Mer2, which also binds nucleosomes (28). We expect this localization immediate adjacent to the hotspot region to be of crucial importance for Mre11 role in promoting DSB formation. With the exception of two separate patches of 5 and 3 amino acids, the deletions in these two Spo11-binding proficient mutants together cover the *mre11-Δ663* deletion, which is Spo11 binding defective, suggesting that only together, they are essential for the binding. This could mean that the two deletions affect two separate domains, each sufficient to promote some Spo11 binding. Noteworthy is the conservation of the Mre11 CTD between *S. cerevisiae* and *C. elegans*, which aligns remarkably well with the requirement of Mre11 for DSB formation in these two organisms, in contrast to higher divergence in other species, where it is dispensable (41).

### Mre11 CTD is needed for the DNA end-bridging activity of Mre11

Mre11 has been shown to tether the DNA ends together, but the domain responsible for this activity is unknown (24,42,43). Since we defined a DNA binding domain within the Mre11 CTD, we reasoned it could be involved in this activity. Indeed, we observed a severe reduction of end-bridging activity in Mre11(1–642) and Mre11(1–663) truncation and a significant reduction in Mre11(1–676), implying a role of the Mre11 CTD in this activity. To our surprise, end-bridging activity was close to normal in the Mre11-Δ668–673 protein, which had a severe DNA binding defect in our assay, indicating bridging activity is independent of robust DNA binding. This data suggests that end-bridging may require only a weak or transient association of Mre11 with DNA. Moreover, we observe a strong stimulation of the Mre11 end-bridging activity by Spo11. Based on our data, it is tempting to speculate that an aspect of end-bridging by Mre11 could have a crucial role in DSB formation as in various steps of DSB repair or replication fork protection in vegetative cells (43). Accordingly, disruption of Mre11 dimerization that coordinates short-range DNA binding sensitizes cells to DNA damage (44).

**Figure 8.**
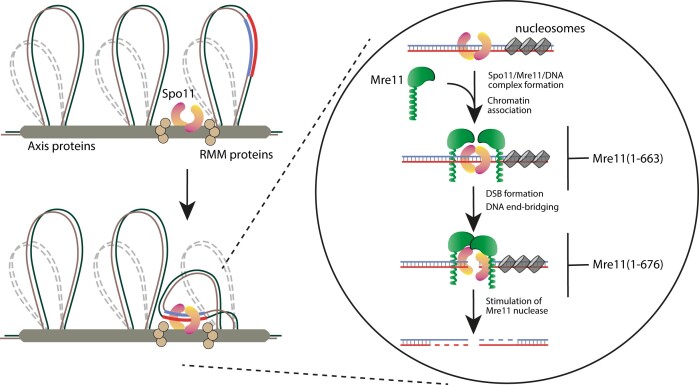
Model for Spo11–Mre11 interaction during meiotic DSB formation. Spo11 and the auxiliary components of the DSB machinery are assembled at the parts of the axial elements. The magnified part concentrates on the recruitment of Mre11 that requires Spo11, a step that is defective in mre11(1–663) and subsequent communication between two Mre11 molecules that may be required to create the proper active cleavage configuration of the Spo11 complex. This configuration may also be in position to nick the correct DNA strand as a requirement for proper 3′-end formation and Spo11-oligonucleotide release.

Based on available data, we propose the following model (Figure 8): Mre11 is essential for Spo11-mediated DSB formation in budding yeast and the observation that Spo11 protein, but not its catalytic function, is required for detectable Mre11 interaction with chromatin before DNA damage, points to a role of Spo11 complex in the recruitment of Mre11. Consistently, we find that Mre11’s autonomous DNA binding activity is not required for its meiotic role. RMM (Rec114, Mei4 and Mer2) proteins assemble in a process requiring Hop1, Red1 and Rec8 (38) upon phosphorylation of Mer2 (45,46). RMM may form globular aggregates (47) acting as a binding platform, which may assist the assembly of the Spo11 and MRX complexes (5,6). Still, all Mre11 mutants that lose *in vitro* interaction with Spo11 are severely defective in DSB formation and spore viability. However, the ability to bind Spo11 is not sufficient for Mre11’s role, since Spo11-interaction proficient *mre11(1–676)* mutant is still defective in DSB formation. Interestingly, Mre11 mutants defective in DSB formation showed a reduction of end-bridging activity. We therefore suggest that one aspect of end-bridging, namely the interaction between Mre11 molecules could be required during DNA breakage. On the other hand, loss of stimulation of mre11(1–676) nuclease activity by N-terminus of Spo11, indicates intricate relationship between Mre11 and Spo11 fragments. Tight coordination between Spo11 and Mre11 might help to direct Mre11 to exclusively nick the DNA strand containing the 5′-tyrosyl ester covalently connecting Spo11. Neither nicking the wrong strand nor nicking both strands has been observed, as the 3′-end is well conserved after cleavage (48). Recognition of the correct strand may require correct orientation relative to Spo11. We propose that the role of the interaction between Spo11 and Mre11 might be to instruct Mre11 to nick the proper strand and promote its correct processing. This might involve a second binding domain within the N-terminus of Spo11 that significantly promotes Mre11 nuclease activity. The intricate interplay between the separate interaction domain in Spo11 further suggest a delicate balance in their molecular interaction. More work is needed to fully unravel the many facets of the Mre11 roles in meiosis. It will be exciting to find and characterise more separation of function mutants, such as *mre11–Δ668–673*. This task will require further dialogue between *in vitro* biochemical, biophysical and *in vivo* genetic and cell biological approaches.

## Supplementary Material

gkae111_Supplemental_File

## Data Availability

All data is contained within the manuscript and/or Supplementary files. ChIP-seq data have been deposited in the Gene Expression Omnibus (GEO) database as GSE253302.
