# Supplementary material for: Physical interaction with Spo11 mediates the localisation of Mre11 to chromatin in meiosis and promotes its nuclease activity
Source: Nucleic Acids Res. 2024 Feb 26;52(8):4328–43. doi: 10.1093/nar/gkae111 (PMC11077076; doi:10.1093/nar/gkae111)
Supplement: gkae111_Supplemental_File [file gkae111_supplemental_file.docx]

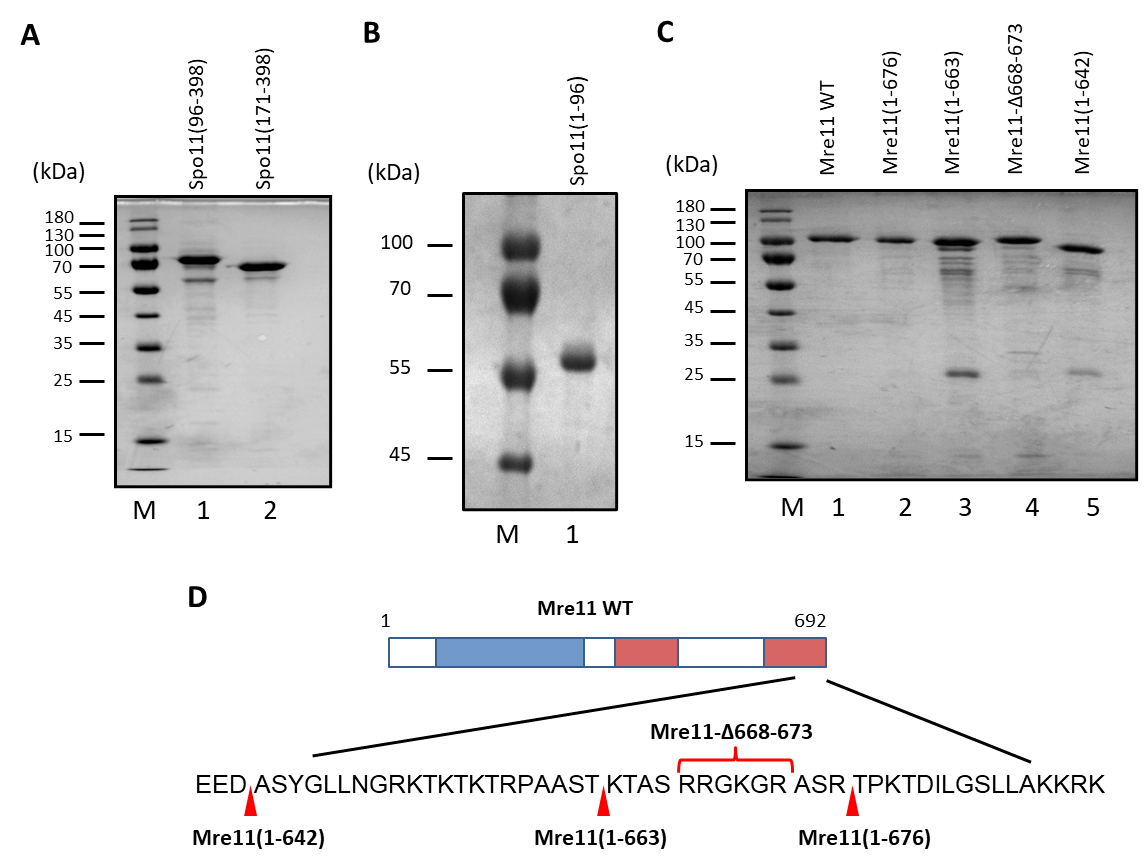


**Figure S1: Proteins used in this study**

One microgram of each protein was resolved by SDS-PAGE and stained with Coomassie blue. **(A)** MBP-His-tagged versions of Spo11(96-398) (lane 1), Spo11 (171-398) (lane 2) and **(B)** Spo11(1-96). **(C)** His-tagged versions of Mre11 WT (lane 1), Mre11(1-676) (lane 2), Mre11(1-663) (lane 3), Mre11-Δ668-673 (lane 4), and Mre11(1-642) (lane 5) proteins. **(D)** Schematical presentation of Mre11 fragments used in the study.

**
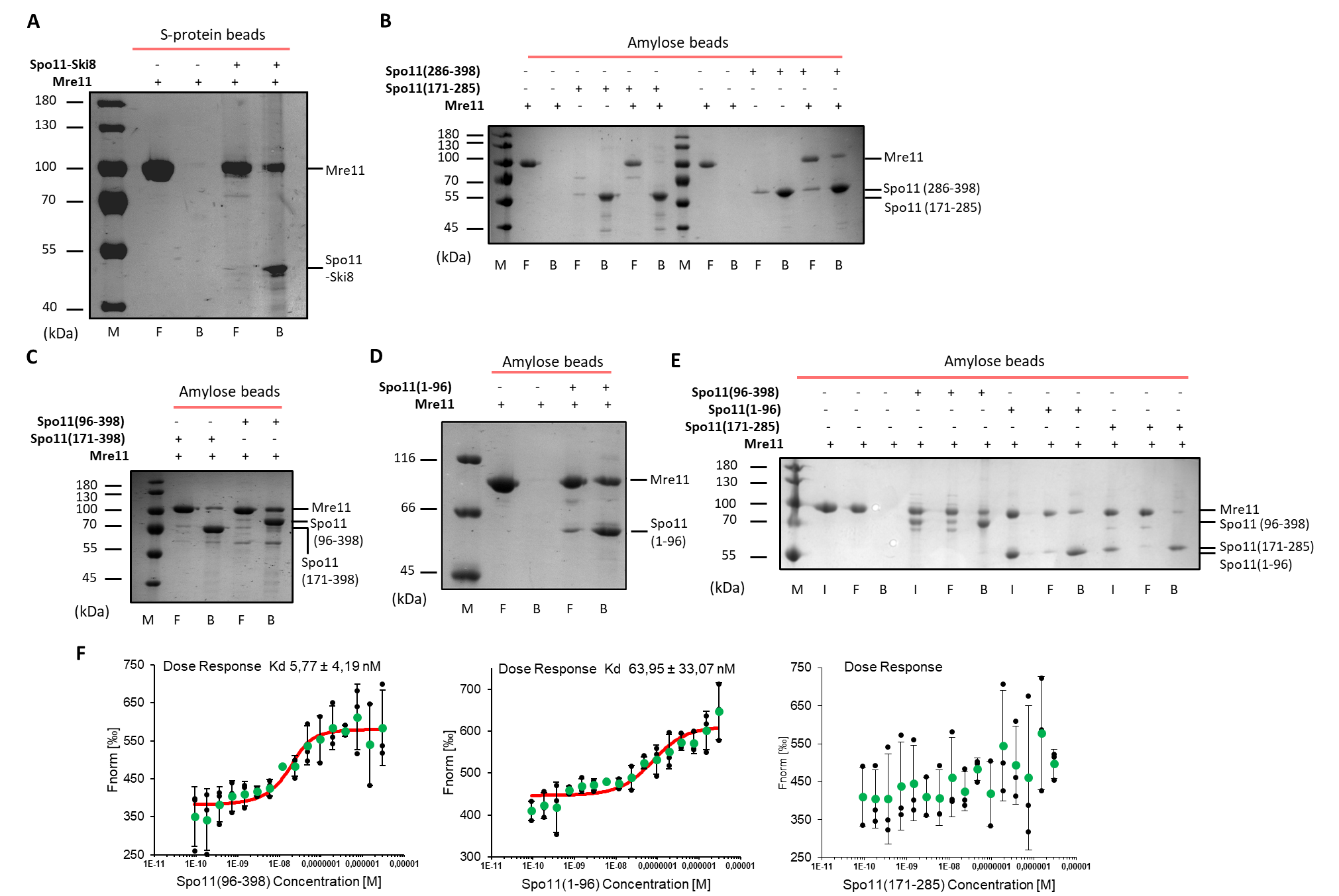
**

**Figure S2: Mapping Mre11 interaction domain within Spo11**

**(A)** Mre11 WT (10 μg) alone or in the presence of Spo11-Ski8 complex (5 μg) were incubated with S-protein beads. The beads were washed and treated with SDS to elute bound proteins. The flow (F) and bead (B) fractions were analysed by SDS-PAGE, followed by Coomassie blue staining. **(B)** Mre11 alone or in the presence of Spo11(171-285) or Spo11(286-398) (10 μg each) was incubated with amylose beads and analysed as in panel A. **(C)** Mre11 interaction with Spo11(96-398) or Spo11(171-398) (10 μg) was analysed as in panel A. **(D)** Mre11 (10 μg) alone or in the presence of Spo11(1-96) (5 μg) was incubated with amylose beads and analysed as in panel A. **(E)** Comparative pull-down of three indicated Spo11 fragments. **(F)** MST measurements to test interaction of fluorescently labelled Mre11 with specified Spo11 fragments. The green circles are means from three replicates, presented as ± s.d. (n=3), while red curve illustrates the fitted binding curve with estimated Kd.

**
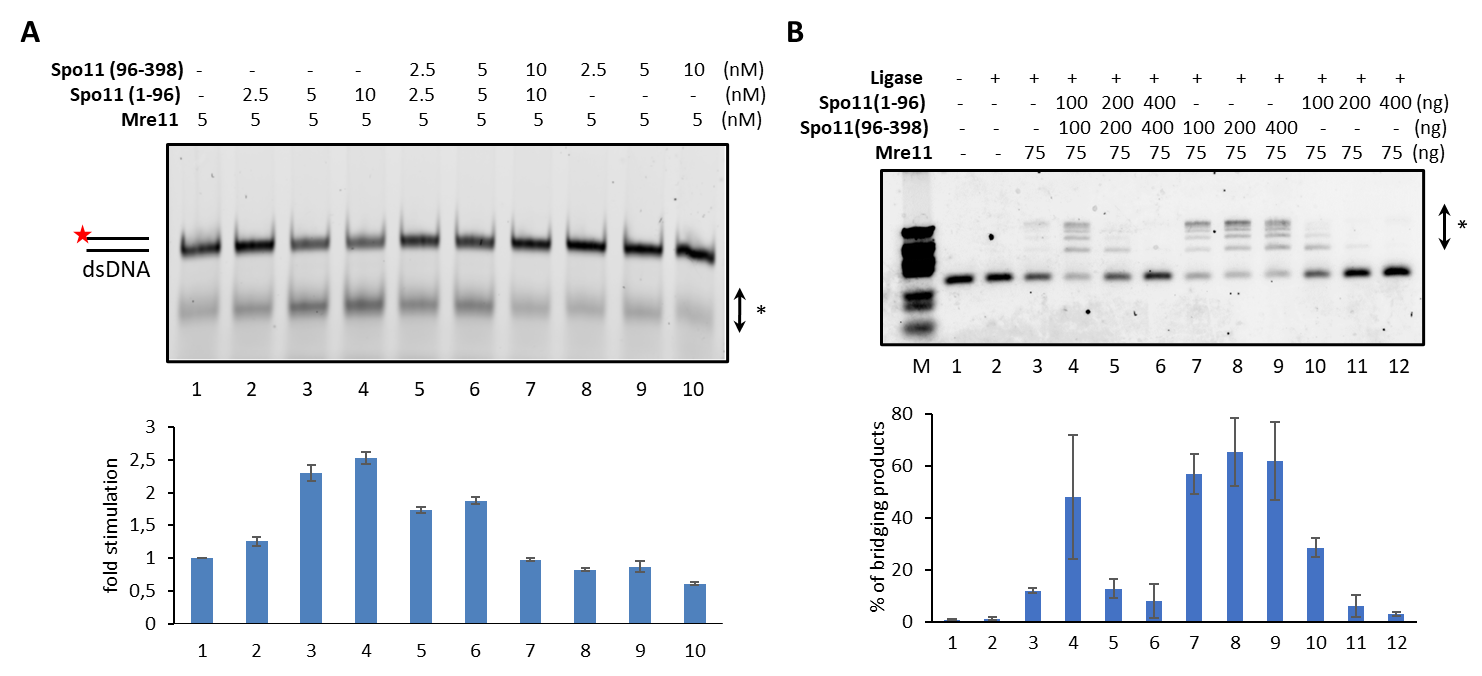
**

**Figure S3: Impact of Spo11(96-398) and Spo11(1-96) on exonuclease and DNA end-bridging activities of Mre11.**

**(A)** Exonuclease activity: Mre11, both in isolation (lane 1) and in the presence of increasing concentrations of Spo11(1-96) (lane 2-4) or Spo11(96-398) (lane 8-10), along with their combination (lane 5-7), were incubated with fluorescently labeled dsDNA (49mer) to assess the exonuclease activity in the presence of MnCl_2_. After incubation, reactions were deproteinised, analysed on 9% native PAGE gel and quantified (n=3). Data are presented as means ± s.d. Asterisk (*) indicates products of the Mre11 nuclease activity. **(B)** DNA end-bridging activity: Mre11, both in isolation (lane 3) and in the presence of increasing concentrations of Spo11(1-96) (lane 10-12) or Spo11(96-398) (lane 7-9), along with their combination (lane 4-6), were incubated with blunt end linearized plasmid in the presence of DNA ligase. Reactions were deproteinised and analysed on 0.7% agarose gel, followed by SYBR Gold staining and quantified (n=3). Data are presented as means ± s.d. * indicates the bridging products. M indicates DNA size marker.


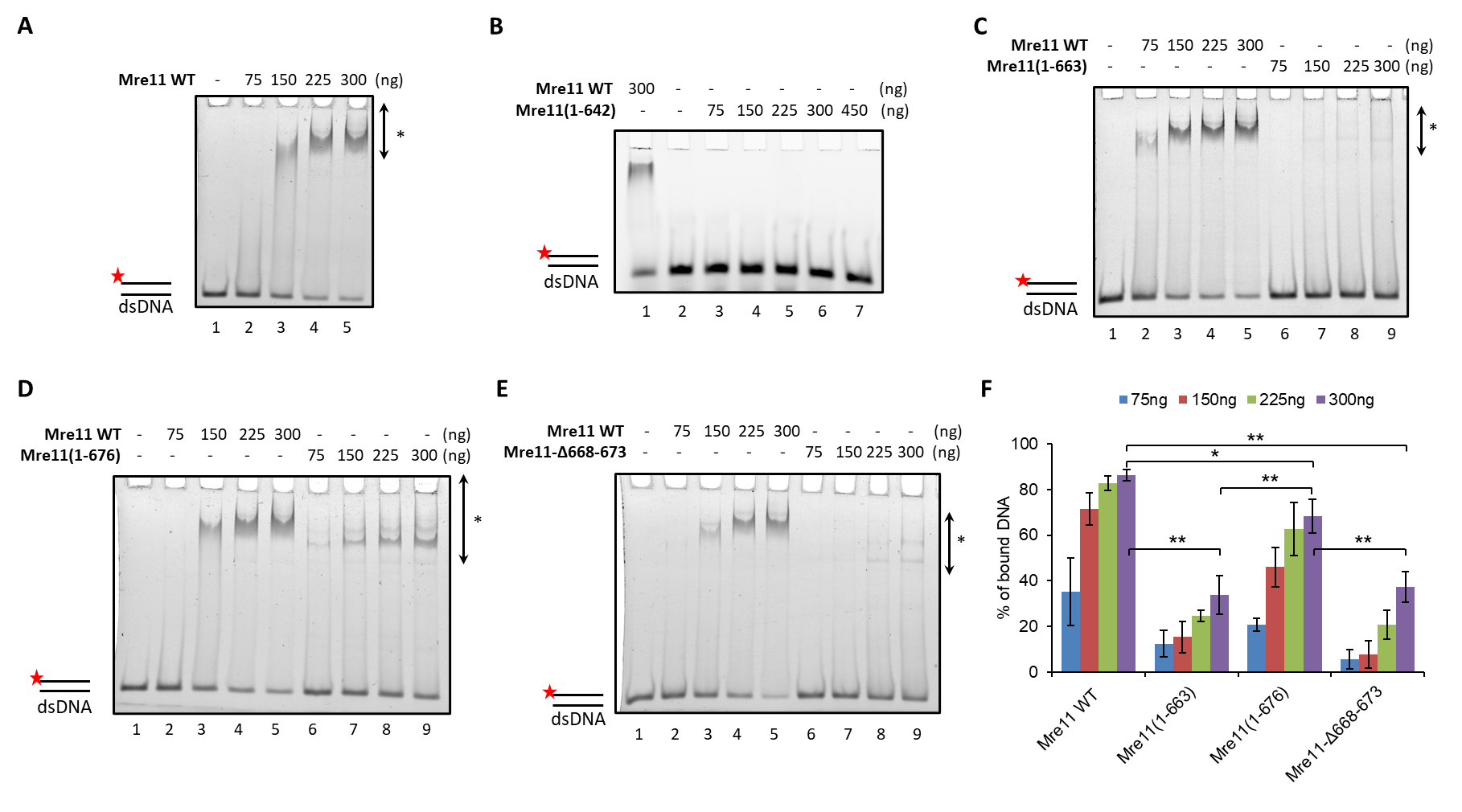


**Figure S4: DNA binding activity of Mre11 variants**

Increasing concentrations of Mre11, (n=3) **(A)**, Mre11(1-642) **(B)**, Mre11(1-663) (lane 6-9), (n=3) **(C)**, Mre11(1-676) (lane 6-9), (n=3) **(D)**, and Mre11-Δ668-673 (lane 6-9), (n=3) **(E)** were incubated with fluorescent dsDNA (49mer) and analysed on 6% native PAGE gel. Asterisks (*) indicate protein-DNA complexes. **(F)** Quantification of DNA binding activity of indicated Mre11 proteins based on three independent experiments; data are means ± s.d. P-values are obtained by Student’s t-test (two-tailed): * p ≤ 0.05; ** p ≤ 0.01.


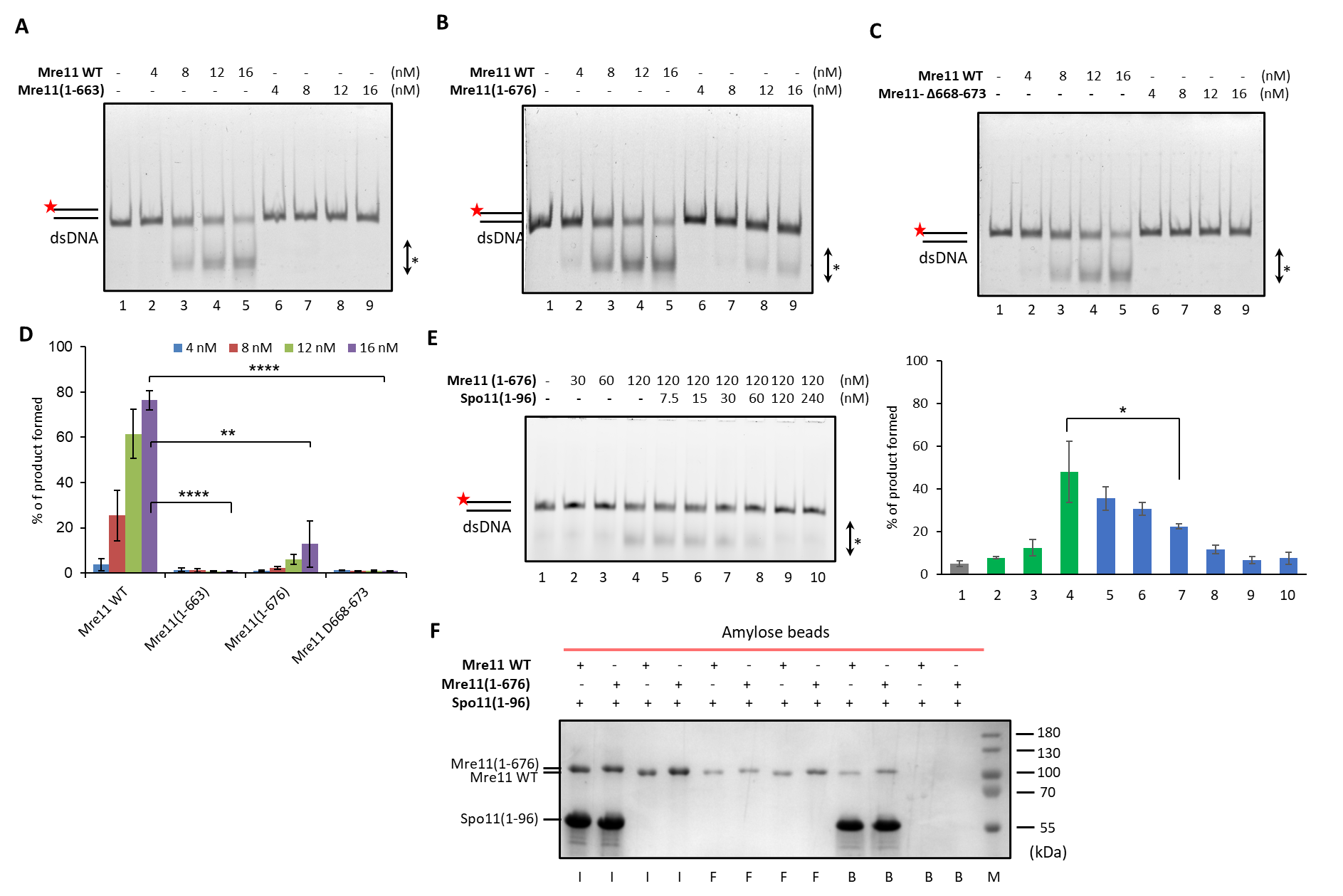


**Figure S5: Exonuclease activity of Mre11 variants**

Increasing concentrations of Mre11 (lanes 2-5) or Mre11(1-663) (lane 6-9) **(A)**, Mre11(1-676) **(B)** and Mre11-Δ668-673 **(C)** were incubated with fluorescent dsDNA (49mer) in the presence of MnCl_2_. Reactions were deproteinised and analysed on 9% native PAGE gel. Asterisk (*) indicates nuclease products. **(D)** Quantification of nuclease products from the indicated Mre11 proteins. Data are presented as means ± s.d. All assays were done in triplicates except for Mre11 WT where n=6. P-values are obtained by Student’s t-test (two-tailed): ** p ≤ 0.01; **** p ≤ 0.0001. **(E)** Exonuclease activity of Mre11(1-676), both in isolation (lane 2-4) and in the presence of increasing concentrations of Spo11(1-96) (lanes 5-10). Right panel: quantification of nuclease activity; data are means ± s.d.; n=3; P-value was obtained by Student’s t-test (two-tailed): * p ≤ 0.05. **(F)** Comparative pull-down of Mre11 WT and Mre11(1-676) with Spo11(1-96) fragment.


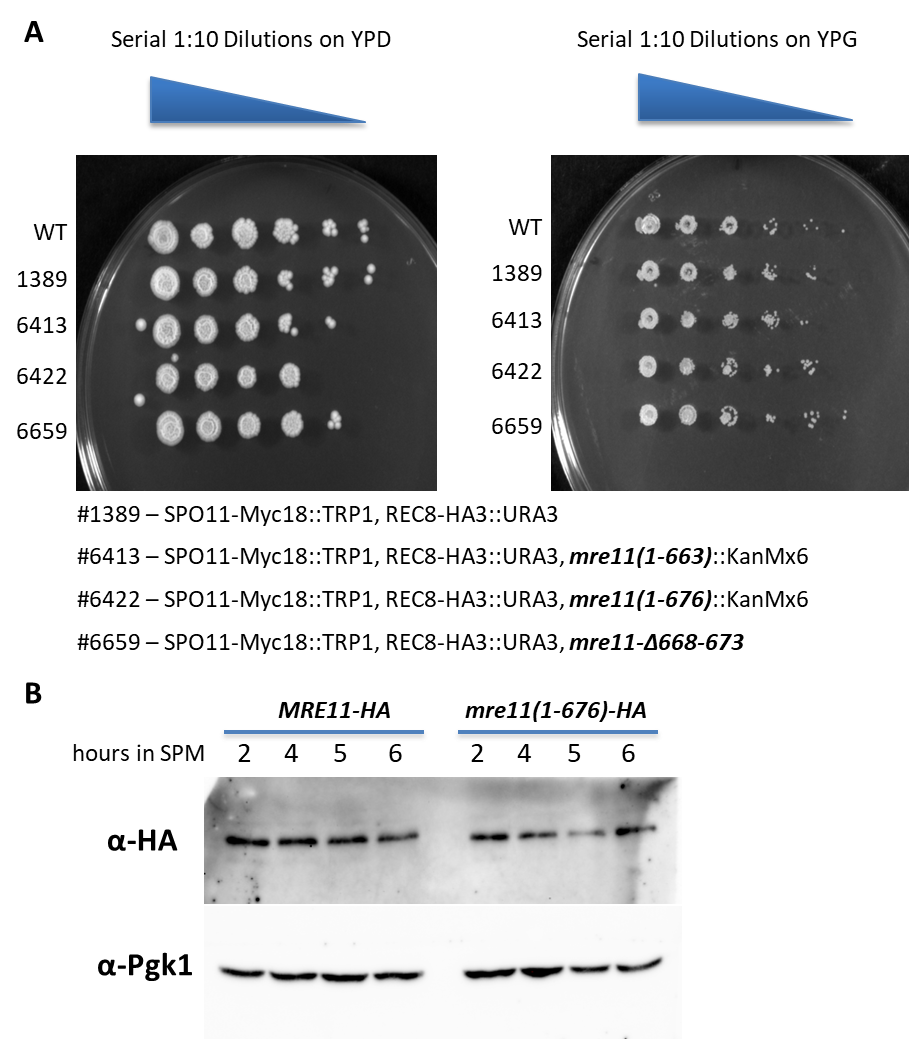


**Figure S6: Vegetative growth of Mre11 variants**

**(A)** Indicated strains were grown overnight on YPD/YPG medium; 10-times serial dilutions of the cultures were plated on YPD/YPG plates and incubated at 30°C for 2-3 days. **(B)** Meiotic cultures from indicated time points after transfer to sporulation medium were collected, and the protein extract from wild-type *MRE11* and *mre11(1-676)* strains were blotted with anti-HA antibody, anti-Pgk1 antibody was used for loading controls.


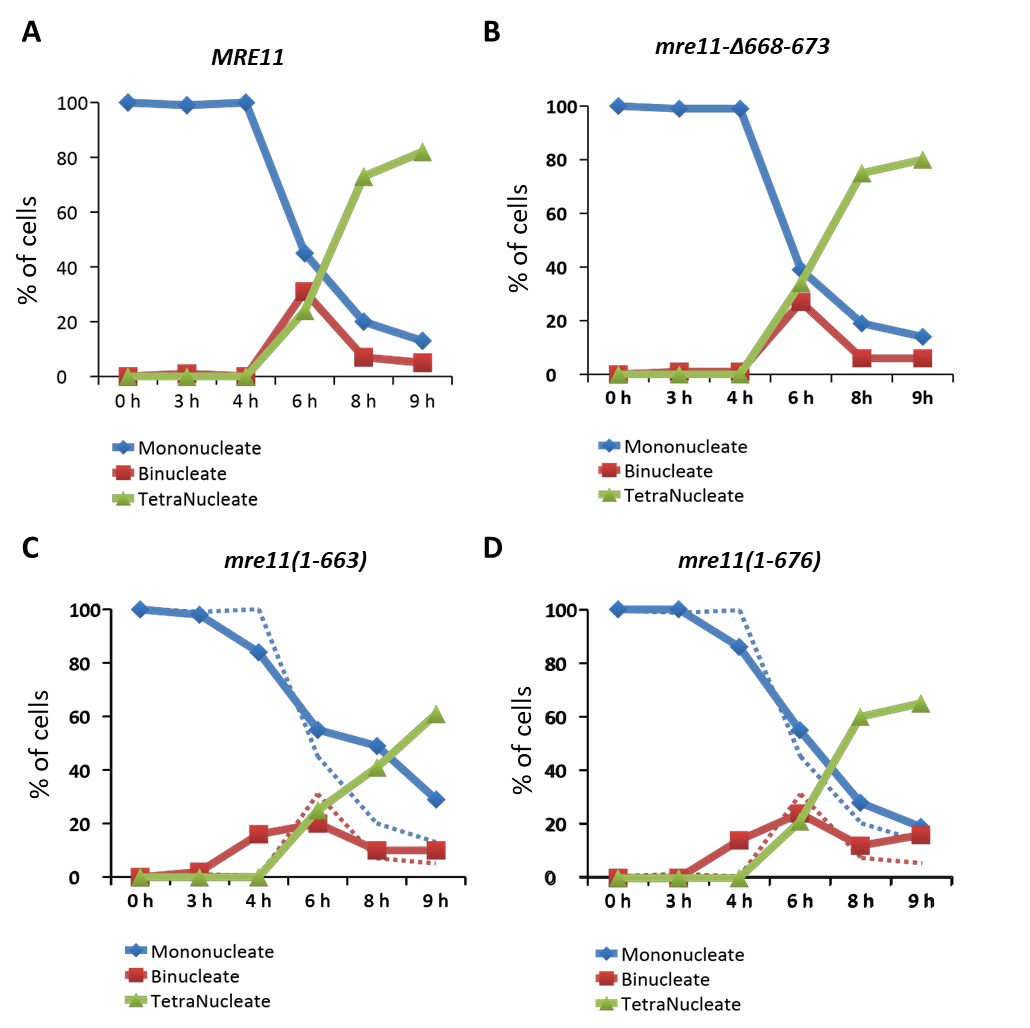


**Figure S7: Mre11 variants complete meiotic divisions**

Meiotic nuclear divisions were analysed by DAPI staining of wild-type *MRE11* **(A)**, *mre11-Δ668-673* **(B)**, *mre11(1-663)* **(C)**, and *mre11(1-676)* **(D)** mutant strains at different time points of meiotic culture. Blue square: cells containing one DAPI-stained nucleus (1n); Red square: cells with two nuclei (2n); Green triangle: cells with four nuclei (4n). Blue and red dotted lines were added to panels C and D for comparison and represent the kinetics of WT *MRE11* shown in panel A.


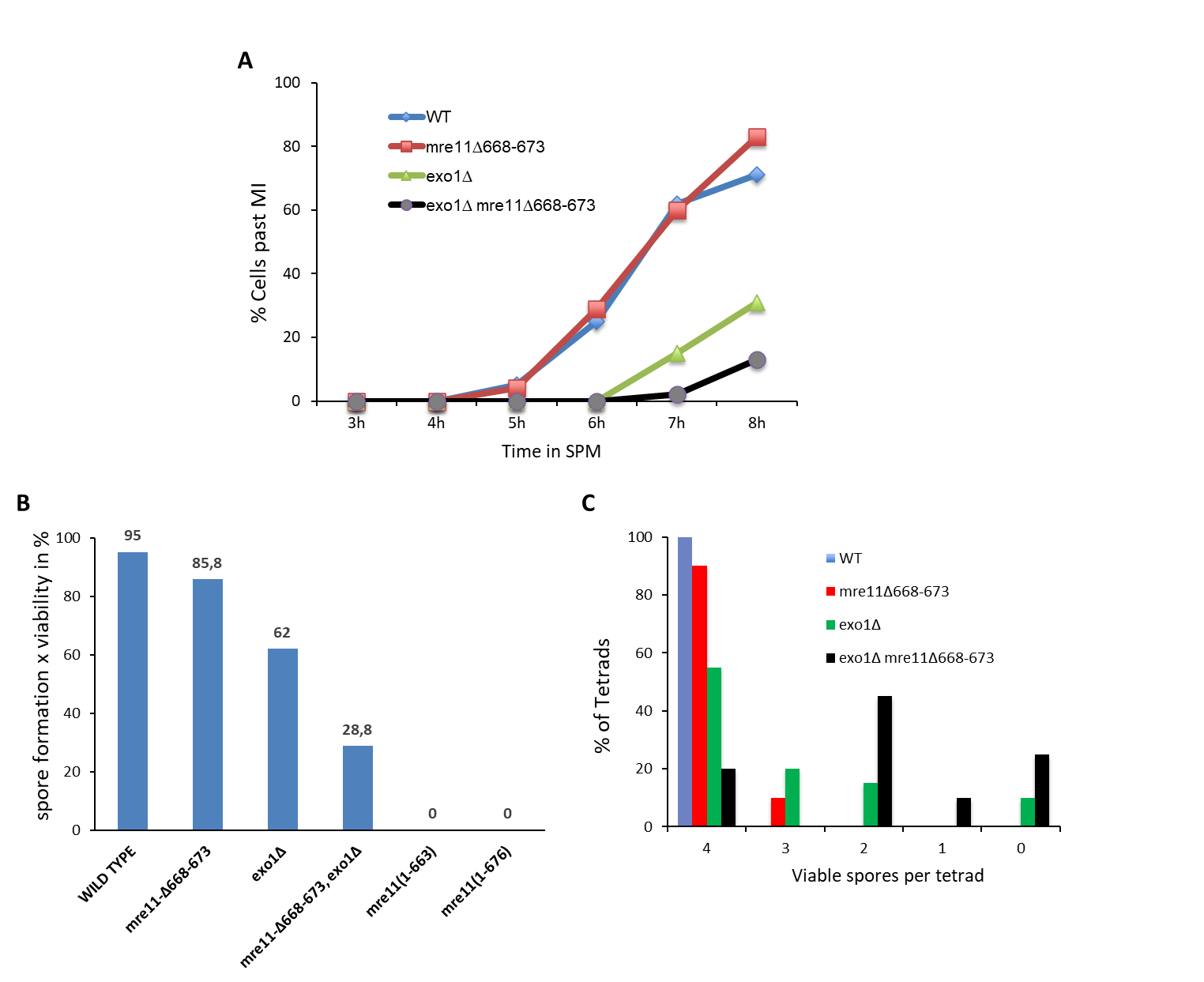


**Figure S8: Viable spore yield and meiotic progression**

**(A)** Cells after Meiosis I (MI), characterized by 2 or 4 nuclei, were quantified after DAPI staining for both single and double mutants of *exo1∆* and *mre11-∆668-673*, alongside the wild type (WT). **(B)** The viable spore yield is calculated as the product of spore formation and spore viability, determined through tetrad dissection. **(C)** Results from the dissection of 20 tetrads each, categorized according to the number of colony-forming spores per tetrad.


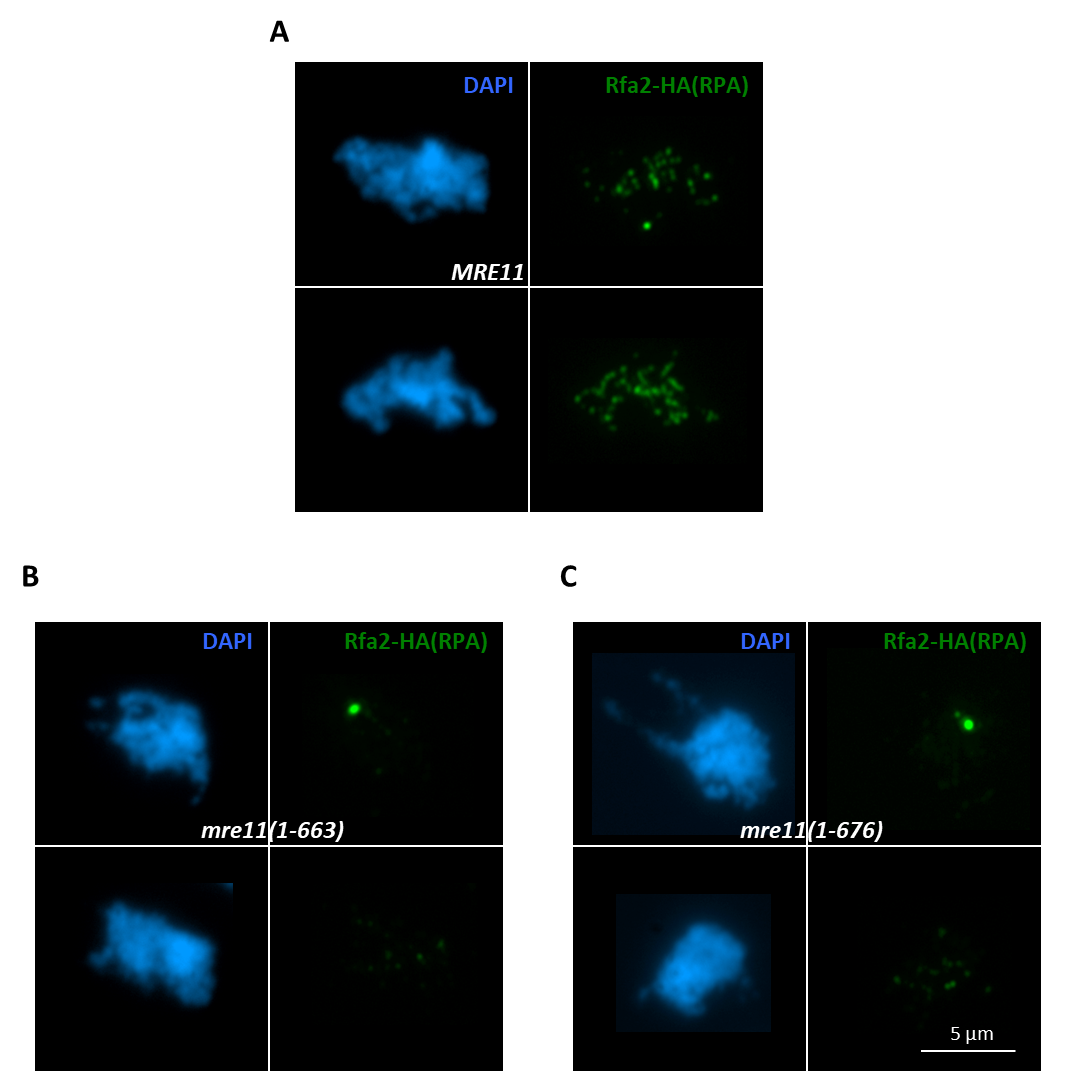


**Figure S9: Rfa2 foci formation in Mre11 variants**

Meiotic nuclear spread of sporulating wild-type *MRE11* **(A)**, *mre11(1-663)* **(B),** and *mre11(1-676)* **(C)** mutant cultures at 4 hours after transfer to SPM media, stained with DAPI and anti-HA antibody for Rfa2.


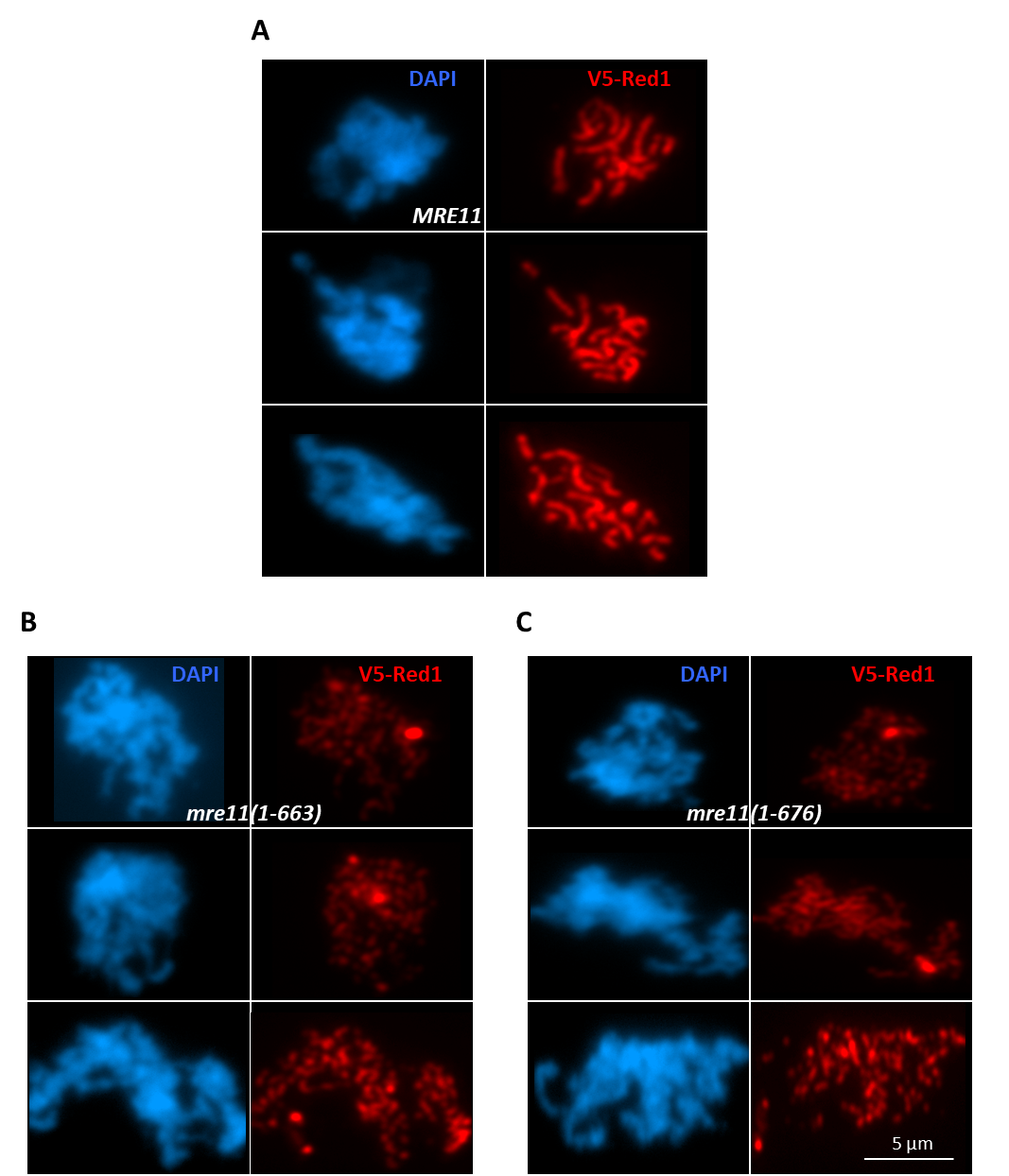


**Figure S10: Chromosomal axes formation in Mre11 variants**

Meiotic nuclear spread of sporulating wild-type *MRE11* **(A)**, *mre11(1-663)* **(B),** and *mre11(1-676)* **(C)** mutant cultures at 4 hours after transfer to SPM media, stained with DAPI and anti-SV5 antibody for Red1.


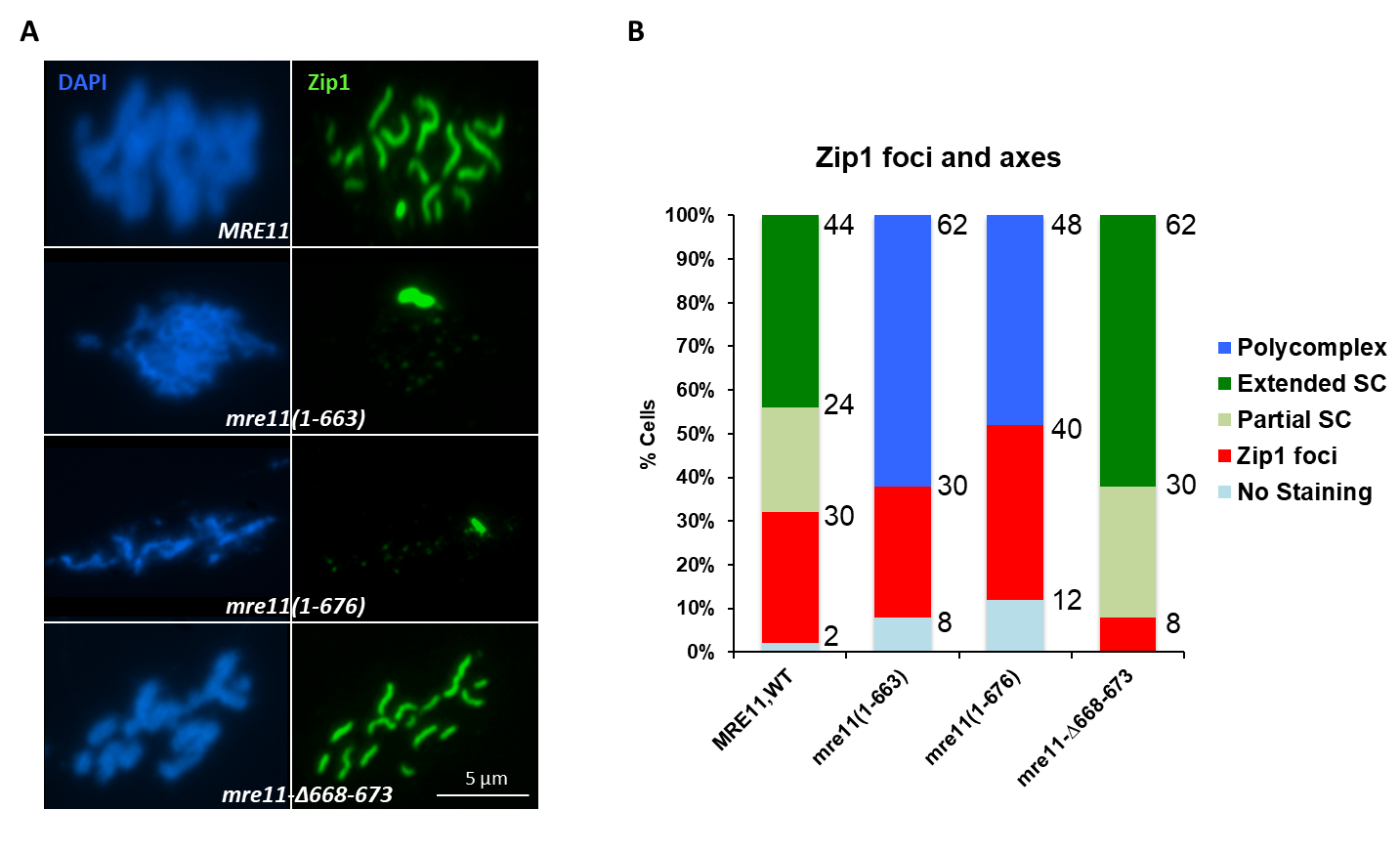


**Figure S11: Synapsis between chromosomes in Mre11 variants at 6 hours in SPM**

**(A)** Meiotic nuclear spread of sporulating wild-type *MRE11*, *mre11(1-663), mre11(1-676),* and *mre11-Δ668-673* mutant cultures at 6 hours after transfer to SPM media stained with DAPI and anti-Zip1 antibody. **(B)** Quantification of 5 categories of nuclei, according to Zip1 staining. Percentages of nuclei containing either polycomplexes only, extended or full SC, partial SC, Zip1 foci or no staining at all. Fifty images per strain were analysed.


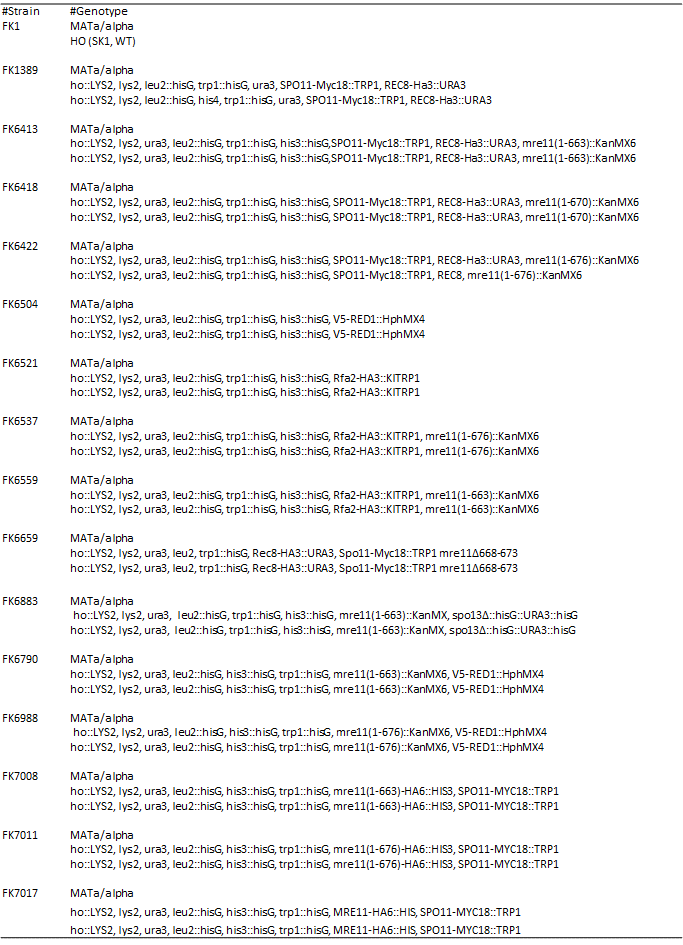
**Table S1: Yeast strains used in this study**


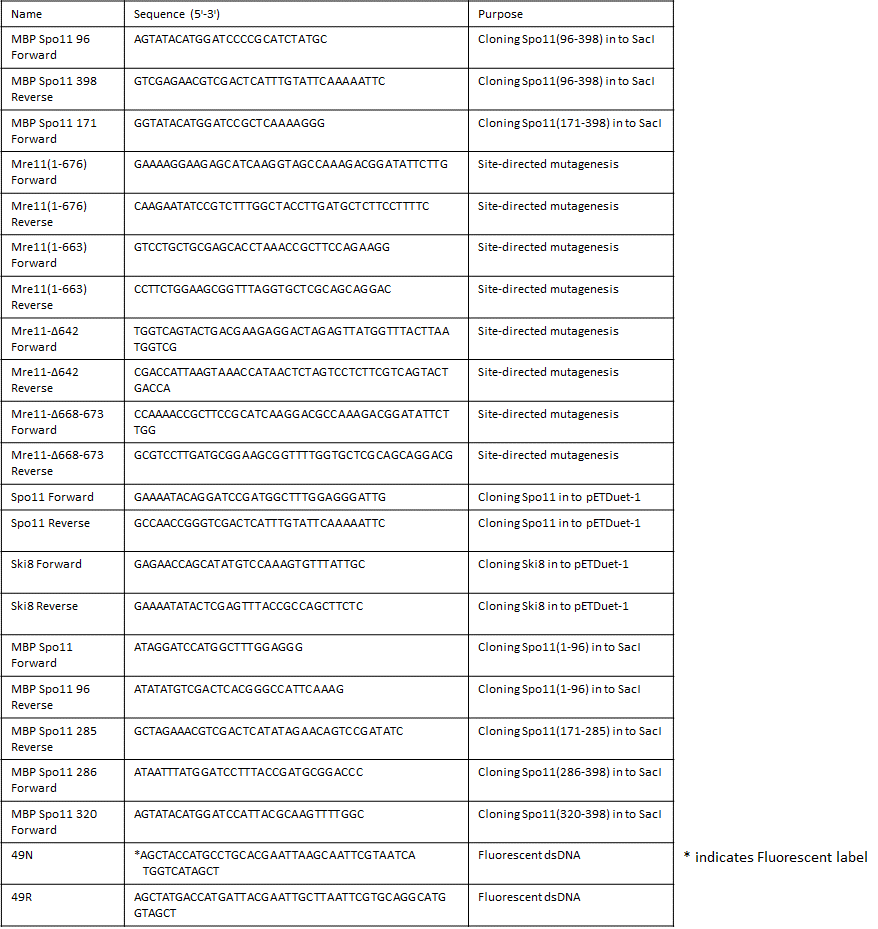
**Table S2: Oligonucleotides used in this study**


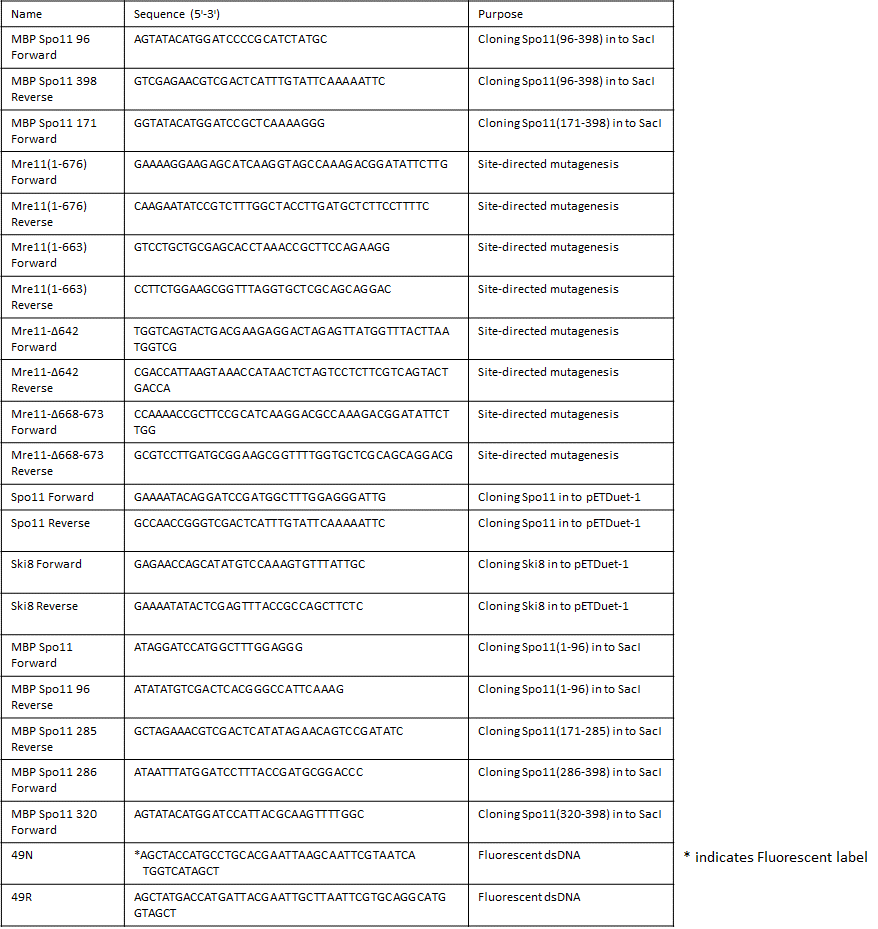
**Table S3: Plasmids used in this study**

| sample.1 | sample.2 | Pearson.corr | Spearman.corr |
| --- | --- | --- | --- |
| Top2 | Mre11 | 0.53 | 0.64 |
| Top2 | Mre11, spo11-135F | 0.43 | 0.56 |
| Top2 | Mre11, spo11∆ | 0.48 | 0.56 |
| Top2 | mre11(1-663) | 0.41 | 0.53 |
| Mre11 | Mre11, spo11-135F | 0.51 | 0.53 |
| Mre11, spo11∆ | Mre11 | 0.5 | 0.52 |
| mre11(1-663) | Mre11 | 0.5 | 0.52 |
| Mre11 | nucleosomes | 0.34 | 0.51 |
| Top2 | mre11(1-676) | 0.4 | 0.49 |
| mre11(1-676) | Mre11 | 0.45 | 0.47 |
| mre11(1-663) | Mre11, spo11-135F | 0.45 | 0.46 |
| Mre11, spo11-135F | nucleosomes | 0.31 | 0.46 |
| Mre11, spo11∆ | Mre11, spo11-135F | 0.44 | 0.45 |
| Mre11, spo11∆ | mre11(1-663) | 0.45 | 0.45 |
| mre11(1-663) | nucleosomes | 0.28 | 0.44 |
| mre11(1-676) | Mre11, spo11-135F | 0.4 | 0.42 |
| mre11(1-676) | mre11(1-663) | 0.41 | 0.42 |
| Mre11, spo11∆ | mre11(1-676) | 0.42 | 0.41 |
| mre11(1-676) | nucleosomes | 0.26 | 0.41 |
| Mre11, spo11∆ | nucleosomes | 0.24 | 0.41 |
| Top2 | nucleosomes | 0.17 | 0.41 |
| Mre11 | nucleosomes reversed | 0 | 0.01 |
| mre11(1-663) | nucleosomes reversed | 0 | 0.01 |
| Mre11, spo11-135F | nucleosomes reversed | 0 | 0.01 |
| mre11(1-676) | nucleosomes reversed | 0 | 0.01 |
| Mre11, spo11∆ | nucleosomes reversed | 0 | 0.01 |
| Top2 | nucleosomes reversed | -0.01 | 0 |
| nucleosomes reversed | nucleosomes | -0.01 | 0 |

**Table S4: Pairwise correlations between genome-wide ChIP signals**


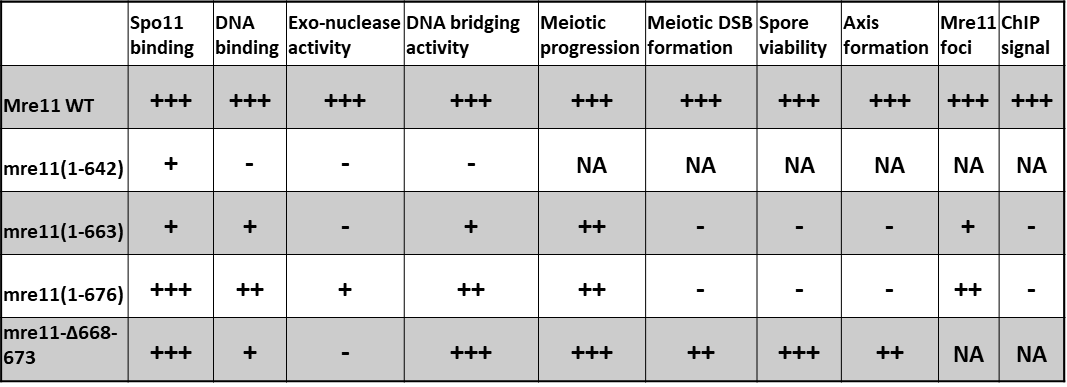


**Table S5: Summary of Mre11 mutant's properties**
